# DeepTYLCV: An interpretable and experimentally validated AI model for predicting virulence of different tomato yellow leaf curl virus strains

**DOI:** 10.1016/j.xplc.2026.101877

**Published:** 2026-04-30

**Authors:** Nattanong Bupi, Hariharan Sangaraju, Duong Thanh Tran, Vinoth Kumar Sangaraju, Hyojin Im, Minkwan Kim, Sukchan Lee, Balachandran Manavalan

**Affiliations:** 1 Celtech Laboratory, Department of Integrative Biotechnology, College of Biotechnology and Bioengineering, Sungkyunkwan University, Suwon, Gyeonggi-do 16419, Republic of Korea; 2 Computational Biology and Bioinformatics Laboratory, Department of Integrative Biotechnology, College of Biotechnology and Bioengineering, Sungkyunkwan University, Suwon, Gyeonggi-do 16419, Republic of Korea

**Keywords:** TYLCV, symptom severity, genome-based prediction, transformer encoder, multi-scale CNN, conventional descriptors

## Abstract

Tomato yellow leaf curl virus (TYLCV) is among the most devastating pathogens affecting tomato production worldwide, with emerging virulent strains increasingly overcoming genetic resistance and triggering severe outbreaks. Traditional field diagnosis, reliant on visual inspection or image-based AI models, remains constrained by symptom dependence, environmental variability, and poor strain-level interpretability. To address these challenges, we introduce DeepTYLCV, a novel deep learning framework for accurate virulence prediction directly from viral-genome-derived open reading frame sequences. We first constructed a comprehensive dataset of globally sourced TYLCV sequences curated by virulence annotations. DeepTYLCV integrates protein language model-based embeddings with optimal concatenated conventional descriptors using a hybrid architecture composed of a transformer encoder and a multi-scale convolutional neural network, enabling effective extraction of both global and local sequence features. Benchmark analyses demonstrate that DeepTYLCV significantly outperforms our previously developed IML-TYLCV model, which was trained on Korean isolates and lacked global generalizability. Importantly, blind predictions on 15 uncharacterized or representative TYLCV isolates were experimentally validated in tomato plants, achieving 100% concordance between model predictions and observed symptom severity. Furthermore, 1D-Grad-CAM++-based interpretability analyses revealed that the model consistently focused on relevant sequence motifs associated with severe strains, offering mechanistic insights into symptom severity. DeepTYLCV is publicly available at https://balalab-skku.org/DeepTYLCV/ and represents a powerful, interpretable, and globally scalable platform for early TYLCV surveillance, resistance monitoring, and strategic disease management in tomato cultivation.

## Teaser

This study introduces DeepTYLCV, a deep learning framework for predicting the virulence of tomato yellow leaf curl virus (TYLCV) directly from viral ORF sequences. The model integrates protein language model embeddings in a hybrid Transformer–MSCNN architecture and outperforms the previous IML-TYLCV model in global prediction tasks. DeepTYLCV predictions were further validated experimentally on 15 viral isolates, achieving 100% concordance. These results highlight the potential of DeepTYLCV as an accurate, interpretable, and scalable tool for TYLCV surveillance and disease management.

## Introduction

Tomatoes, as one of the world’s most consumed and economically vital vegetables, have their agricultural output directly tied to their healthy growth (Collins et al., 2022). However, this production is under constant assault from various pathogens, among them the tomato yellow leaf curl virus (TYLCV; species *Begomovirus coheni*, genus *Begomovirus*) (Li et al., 2022). As the most common and destructive virus, TYLCV poses a serious threat by disrupting normal plant growth and causing telltale symptoms such as leaf yellowing and curling (Prasad et al., 2020). Importantly, TYLCV has different strains that cause different levels of disease severity (Marchant et al., 2023). The classic “Israel” strain is known for triggering severe symptoms, including stunted growth, leaf curling and yellowing, and, in some cases, the early loss of flowers and fruits (Navot et al., 1991; Hanssen et al., 2010; Navas-Castillo et al., 2011). Conversely, the “mild” strain tends to cause only slight leaf curling and yellowing, allowing infected plants to continue growing with less impact on yield (Antignus and Cohen, 1994). Alarmingly, the more severe strains have become widespread in recent years. These virulent types have even overcome genetic resistance in some tomato varieties, such as those carrying the *Ty*-1 gene, resulting in serious outbreaks despite significant breeding efforts for tolerance (Torre et al., 2018; Jammes et al., 2023; Koeda and Kitawaki, 2024). The accelerating global threat posed by TYLCV strains highlights the urgent need for a proactive surveillance strategy (Metsky et al., 2022).

Conventional field diagnosis relies on growers’ visual inspection and experience, making it subjective, fatigue prone, and poorly suited to early detection (Mahlein, 2016; Demilie, 2024; George et al., 2025). To improve field diagnostic speed and scalability, AI-based image recognition tools have been developed to classify TYLCV from leaf snapshots and are increasingly deployed in agricultural settings. Several methods have been reported in the literature to identify tomato leaf disease from images, advancing the field significantly (Shoaib et al., 2022; Shmaryahu et al., 2024; Chen et al., 2025). However, these approaches remain symptom dependent and thus insensitive to pre-symptomatic or asymptomatic infections, when intervention is most effective. Phenotypes are non-specific and confounded by abiotic stress, lighting variations, and co-infections, which has resulted in weakened diagnostic sensitivity and specificity (Bostock et al., 2014; Tanner et al., 2022). Image-based models are data sensitive and require frequent retraining to adapt across cultivars, geographies, or environments (Cubero et al., 2024). Critically, they offer limited biological interpretability, providing neither reliable strain-level discrimination nor mechanistic insight into disease severity (Karthikeyan et al., 2025; Kondaveeti and Simhadri, 2025).

Genome-based prediction offers a significant advantage over traditional image-based methods in the early and accurate identification of TYLCVs (Silva et al., 2017; Sukhorukov et al., 2022). Unlike image-based tools, genome-based tools can directly detect mild and severe strains from the primary sequence information. Moreover, these models are generalizable, remaining accurate across diverse environments and cultivars, as they are unaffected by external factors such as lighting or abiotic stress. This makes genome-based diagnostics ideal for proactive surveillance, resistance monitoring, and strategic disease management. Specifically, genome-based diagnostics streamline resistance monitoring by identifying target viruses and characterizing the virulence of rapidly evolving isolates, thereby reducing reliance on conventional bioassays. In our previous work, we developed an IML-TYLCV predictor based on Korean TYLCV strains (Bupi et al., 2023). However, a critical limitation remains: this localized model cannot be universally applied to global TYLCV sequences. To address this gap and maximize utility for researchers worldwide, it is essential to build an expanded, global genome-based model using strains collected from diverse geographic regions. Such a universal predictor will be an invaluable tool for global surveillance and strategic disease management, informing researchers and growers globally on emerging disease severity.

In this study, we introduce DeepTYLCV, a novel AI framework for predicting TYLCV symptom severity directly from open reading frames (ORFs) extracted from viral genomes. Comprehensive benchmarking demonstrates that DeepTYLCV substantially outperforms the existing IML-TYLCV model and addresses its limitations in handling the globally genetic diversity of TYLCV isolates. Importantly, blind prediction experiments with independent wet-lab validation confirm both the robustness and the translational potential of the framework. Beyond accurate prediction, DeepTYLCV provides interpretable biological insights into the sequence features associated with TYLCV virulence, thereby advancing our mechanistic understanding of symptom severity. By pinpointing severity-associated genomic regions, the proposed method may help identify candidate targets for resistance breeding and could serve as a useful resource for the seed industry in developing TYLCV-resistant tomato cultivars through targeted molecular selection.

## Results and discussion

### Framework and construction of DeepTYLCV

**Figure 1:**
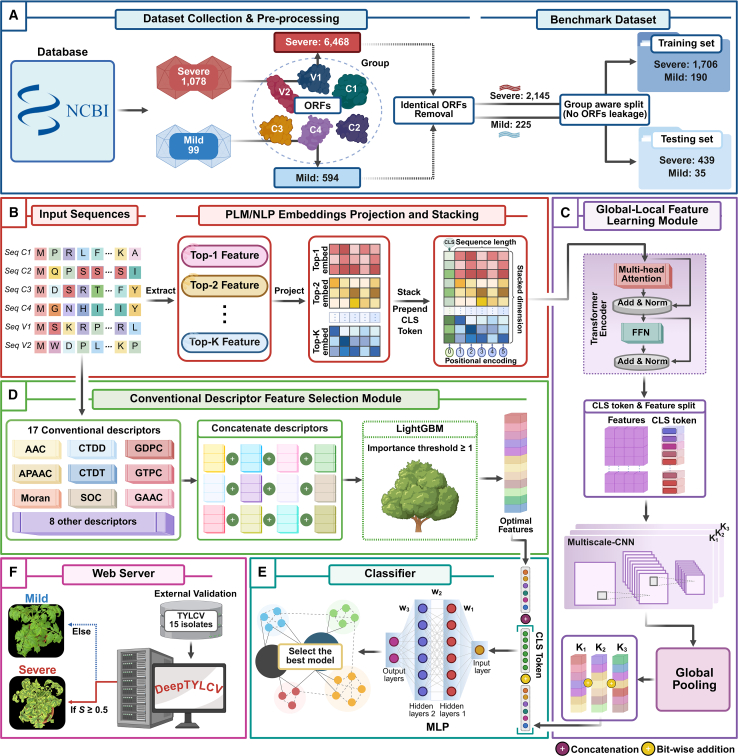
The workflow overview for DeepTYLCV, which consists of five integrated modules. **(A)** Dataset collection and pre-processing: 1177 TYLCV genomes (1078 severe and 99 mild) were retrieved from NCBI, translated into six open reading frames (ORFs) (C1–C4, V1, and V2), and divided into group-aware training and testing sets. **(B)** The feature processing (FP) module, including PLM/NLP embedding projection and stacking: ORFs were encoded using 12 PLMs and three NLPs, and Top-*K* PLM/NLP-based embeddings were projected and stacked along the feature dimension to retain contextual information, with the CLS token prepended to the sequence length dimension to capture global information. **(C)** The global–local feature learning module, comprising the transformer encoder (TE) and multi-scale convolutional neural network (MSCNN), captures long-range dependencies and local motifs through hybrid transformer-convolutional layers. **(D)** The conventional descriptor feature selection module, comprising 17 physicochemical and composition-based descriptors, was used, and the optimal features were selected with LightGBM to extract the most discriminative properties and eliminate redundancy. **(E)** Classifier: the learned feature representations from the TE and MSCNN were concatenated with optCCDs, which were then fed to the multi-layer perceptron (MLP) to classify as severe or mild. **(F)** Web server: the best classifier module (severe if *S* ≥ 0.5, mild otherwise), which is deployed as a user-friendly web server.

To predict TYLCV symptom severity from viral coding sequences, we developed DeepTYLCV, an interpretable deep learning framework that integrates complementary sequence representations with local-global feature learning (Figure 1). DeepTYLCV combines protein language model (PLM)-based embeddings and natural language processing (NLP)-based embeddings derived from large-scale pre-trained models with optimal concatenated conventional descriptors (optCCDs) to capture the diverse properties of TYLCV ORFs (Pham et al., 2024; Tran et al., 2025a). Specifically, the framework employs a hybrid architecture that combines transformer-based global contextual dependencies with a multi-scale convolutional neural network to extract local sequence motifs, along with an optimized physicochemical feature selection, enabling the model to effectively learn both distributed contextual signals and fine-grained, virulence-associated patterns. To enhance interpretability, the framework also includes an explainability module that maps model importance scores onto the viral genome.

### Systematic benchmarking of PLM and classical NLP embeddings for TYLCV severity prediction

To rigorously evaluate sequence representation strategies for TYLCV severity prediction, we conducted a comprehensive comparison of 15 PLM/NLP-based embeddings. All models were first assessed via five-fold cross-validation and then validated on an independent test set. Notably, these experiments utilized our framework without incorporating the optCCDs to isolate its impact on predictive performance.

**Figure 2:**
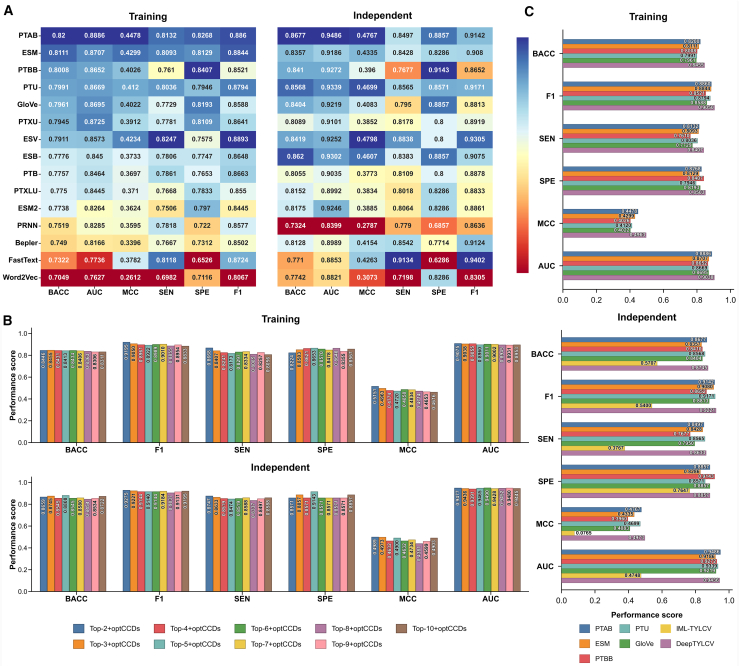
Comparative evaluation of baseline models, DeepTYLCV, and existing methods. **(A)** Performance comparison of individual PLM/NLP-based embeddings on the training and independent test sets. **(B)** Performance of hybrid models integrating PLM/NLP-based embeddings with optCCDs selected by LightGBM, evaluated on both training and independent sets. **(C)** Performance comparison of DeepTYLCV with the top five baseline models and the existing IML-TYLCV method.

Results demonstrated that PLM-based embeddings substantially outperformed classical NLP-based embeddings (Figure 2A). Among the models evaluated, ProtTrans-ALBERT-BFD (PTAB) emerged as the top performer on the training set, achieving a balanced accuracy (BACC) of 0.820, an area under the receiver operating characteristic curve (AUC) of 0.8886, a Matthews correlation coefficient (MCC) of 0.4478, and an F1 score (F1) of 0.886. Compared with other embedding strategies, PTAB improvements ranged from 0.89% to 11.51% in BACC, 1.79% to 12.59% in AUC, and 1.79% to 18.66% in MCC. These results highlight that PTAB captures biologically meaningful sequence patterns associated with TYLCV virulence more effectively than other embeddings. Other PLM-based models, including ESM-1 (ESM), ProtTrans-BERT-BFD (PTBB), and ProtTrans-T5-UniRef50 (PTU), also achieved strong performance, but consistently remained below PTAB across the major evaluation metrics. In contrast, classical NLP-based models such as FastText (achieved 0.7322 in BACC, 0.7736 in AUC, and 0.3782 in MCC) and Word2Vec (achieved 0.7049 in BACC, 0.7627 in AUC, and 0.2612 in MCC) exhibited significantly inferior performance, underscoring their limited ability to capture the complex, context-dependent genomic signals necessary for symptom severity prediction.

To further validate model generalization, we assessed all 15 PLM/NLP-based embeddings on an independent test set. The relative performance rankings observed during cross-validation were largely preserved. PTAB once again achieved the highest predictive performance, with a BACC of 0.8677, AUC of 0.9486, MCC of 0.4767, sensitivity of 0.8497, specificity of 0.8857, and F1 of 0.9142, the best across all embeddings tested. This strong agreement between cross-validation and independent test results underscores PTAB’s robust generalization and resistance to overfitting. When compared with the second-best model (ESM), PTAB exhibited 3.2% improvement in BACC (0.8677 vs. 0.8357), further emphasizing its superior discriminatory power. Conversely, shallow NLP-based embeddings continued to perform poorly on the independent test set, confirming that they are insufficient for modeling informative genomic features underlying TYLCV severity. Overall, these results demonstrate that pre-trained PLMs, especially PTAB, are more powerful for TYLCV prediction than classical embedding strategies.

Importantly, this benchmarking analysis also provided the conceptual basis for the design of DeepTYLCV. Although PTAB was the single best-performing embedding, no individual embedding can be assumed to capture all virulence-relevant sequence information. We therefore next investigated whether integrating complementary feature types, including PLM-based embeddings, NLP-based embeddings, and optCCDs, could further improve predictive robustness and generalizability within the DeepTYLCV framework.

### Development of DeepTYLCV

To systematically develop an accurate and generalizable model for TYLCV severity prediction, we first constructed a diverse feature space comprising multiple sequence encoding strategies. Based on the performance of the baseline models described in the previous section, embeddings were ranked in descending order according to BACC. Using this ranking, we progressively integrated the top 2, top 3, top 4, and up to the top 10 embeddings, each further combined with the optCCDs. Notably, among the optCCDs, the composition of *k*-spaced amino acid pairs was the dominant descriptor, contributing 90 feature dimensions (22.84%), indicating a strong ability to capture pairwise amino acid relationships critical for classification. Distribution in “composition/transition/distribution” (CTD) descriptors and quasi-sequence order followed, with 50 and 45 feature dimensions (12.69% and 11.42%), respectively (Supplemental Figure 1), highlighting the importance of physicochemical property transitions and sequence-order information in discriminating between mild and severe isolates. Each composite feature set was then used to train an independent DeepTYLCV model, which was subsequently evaluated on the independent test set to assess performance consistency and generalization capability.

Figure 2B illustrates that the Top-2+optCCDs to Top-6+optCCDs configurations achieved superior performance compared with the remaining models. Among them, the Top-3+optCCDs, which integrates PTAB, ESM, and PTBB with optCCDs, achieved the best performance, with a BACC of 0.8455 on the training set and 0.8745 on the independent test set. This result suggests that integrating PLM/NLP-based embeddings with handcrafted features creates a synergistic feature space that captures both deep contextual sequence patterns and biologically meaningful properties. This hybrid-feature approach enhances model robustness and generalizability, enabling more accurate discrimination between mild and severe TYLCV isolates compared with any single-feature model. In contrast, models utilizing the Top-7+optCCDs to Top-10+optCCDs showed inconsistent performance between the training and the independent test sets, likely due to the inclusion of redundant or noisy features that degrade model generalization. Based on these results, we selected the Top-3+optCCDs as the final configuration for DeepTYLCV, which provided the best balance of predictive accuracy, generalizability, and feature efficiency.

To further examine the biological limits of DeepTYLCV, we analyzed the misclassified isolates in the independent test set by calculating misprediction error rate across the six canonical ORFs (Supplemental Table 1). This analysis revealed that most classification errors originated from the V1 and V2, which exhibited error rates of 37.8% and 19.1%, respectively. This pattern is biologically plausible and consistent with the known functional role of these proteins. Beyond its structural role, V1 is critical for whitefly-mediated transmission and may be further influenced by frequent recombination events (Saleem et al., 2016; Czosnek et al., 2017; Mondal et al., 2019; Yogindran et al., 2021), whereas V2 is a multi-functional pathogenicity factor primarily involved in immune evasion through the suppression of host RNA silencing and facilitates viral movement (Rishishwar and Dasgupta, 2019; Zhao et al., 2020). As a central component of the host–virus interaction network, V2 is under strong selective pressure to counteract host defenses, resulting in increased sequence variability. This variability likely limits the model’s ability to identify these ORFs and may contribute to the observed misclassifications. Together, these findings indicate that prediction errors are observed in biologically dynamic regions of the TYLCV genome, underscoring both the complexity of symptom severity prediction and the biological relevance of the DeepTYLCV framework.

### Comparison of DeepTYLCV with top-performing baseline models and IML-TYLCV

To demonstrate the superiority of DeepTYLCV, we compared its performance against five top-performing baseline models trained using individual embeddings, namely PTAB, ESM, PTBB, PTU, and GloVe. On the training set (Figure 2C), DeepTYLCV achieved the highest scores across all major metrics. Notably, it outperformed the baseline models with improvements in the range 2.55%–4.94% in BACC, 1.52%–3.86% in AUC, and 4.85%–9.41% in MCC, highlighting the advantage of hybrid-feature integration. DeepTYLCV also achieved the lowest false discovery rate (FDR = 0.0182), indicating that its decision boundary is well calibrated and effectively separates mild and severe strains.

Evaluation on the independent test set (Figure 2C) further confirmed its strong generalization. DeepTYLCV maintained the highest performance, with improvements of 0.68%–3.88% in BACC and 2.06%–10.13% in MCC compared with individual baseline models. In addition, DeepTYLCV minimized both false negatives (60 out of 439 severe samples) and false positives (4 out of 35 mild samples), an important property for reducing the risk of overlooking severe isolates. Within this same comparative analysis, DeepTYLCV significantly outperformed our previous model, IML-TYLCV, across all evaluation metrics. Since IML-TYLCV was trained exclusively on Korean isolates, its performance degraded substantially when applied to geographically diverse TYLCV sequences, underscoring the limitations of region-specific models. To further highlight the limited global generalizability, we evaluated IML-TYLCV separately on Korean and non-Korean isolates within the independent test set (Supplemental Figure 2). The model showed a clear difference in performance between the two groups. On Korean isolates, IML-TYLCV achieved a BACC of 0.7750 and an MCC of 0.5185. However, when applied to non-Korean isolates, performance declined significantly to a BACC of 0.5694 and an MCC of 0.0656, representing performance decreases of 20.56% and 45.29%, respectively. This significant performance gap clearly demonstrates the model’s limited transferability beyond Korean genomes. In contrast, DeepTYLCV leverages multiple PLM/NLP-based embeddings with conventional descriptors to capture broader sequence diversity and reduce the effect of dataset shift, resulting in a robust and globally applicable TYLCV severity prediction.

Beyond these standard classification metrics, we also evaluated the predictive calibration by comparing the log-loss distribution of DeepTYLCV against the best single-embedding baseline model (PTAB) on the independent test set. Paired bootstrap testing (*n* = 10 000 resamples) demonstrated that DeepTYLCV achieved a statistically significant reduction in overall log-loss across all samples (*p* < 0.05) (Supplemental Figure 3). Stratified analysis further revealed that this improvement was primarily driven by superior performance on positive (severe) samples, for which DeepTYLCV showed a highly significant gain in predictive confidence (*p* < 0.001). Conversely, the log-loss distribution for negative (mild) samples remained comparable to the baseline model (*p* = 0.447). These results indicate that the integration of multiple complementary feature types in DeepTYLCV improves not only discrimination but also the reliability of probability estimates, particularly for severe isolates. This is practically relevant because well-calibrated predictions can support more confident prioritization of candidate severe isolates for downstream experimental validation. In resource-intensive approaches, such as infectivity assays, symptom evaluation, and host–virus interaction studies, this improved probabilistic reliability can help them to allocate experimental effort more efficiently.

Collectively, these results establish DeepTYLCV as a high-performance and globally transferable framework for plant viral pathogenicity prediction. Its architecture highlights the value of hybrid-feature integration in capturing biologically relevant sequences, making it a powerful tool for large-scale TYLCV surveillance, resistance monitoring, and precision agriculture.

### Evaluating the impact of additional small ORFs (V3 and C5) on predictive performance

While the current DeepTYLCV framework is based on the six canonical TYLCV ORFs, recent molecular studies have identified additional small ORFs in geminiviruses, including V3 and C5, that may contribute to viral infectivity and symptom exacerbation (Gong et al., 2021; Zhao et al., 2022). To investigate whether these small ORFs could further improve predictive performance, we developed an expanded eight-ORF version of model. This extended framework was trained and evaluated using the same optimization pipeline as the original six-ORF architecture. We first assessed individual PLM- and NLP-based feature embeddings (Supplemental Figure 4A) and then constructed hybrid models by combining the top-ranked PLM/NLP-based embeddings with optCCDs (Supplemental Figure 4B).

Our results demonstrated that incorporating V3 and C5 did not improve overall predictive performance. The highest-performing eight-ORF hybrid-feature model (Top-2 PLMs+optCCDs) achieved a BACC of 0.8349 and an MCC of 0.4669 during training. On the independent test set, this expanded model achieved a BACC of 0.8607 and an MCC of 0.4579. In contrast, the final six-ORF DeepTYLCV model demonstrated superior generalization, achieving a BACC of 0.8745 and an MCC of 0.4973 on the independent test set. These findings indicate that the addition of V3 and C5 does not provide a measurable predictive benefit within the current modeling framework.

This lack of improvement may be explained by the highly compact and overlapping organization of the TYLCV genome. Because small ORFs such as V3 and C5 are embedded within or overlap with major coding regions, part of their sequence variation may already be captured by the PLM/NLP-based embeddings from the six canonical proteins. Consequently, introducing V3 and C5 as separate inputs may increase feature complexity without contributing sufficiently additional information for severity classification. Ultimately, these findings underscore a critical modeling truth: biological relevance does not guarantee predictive power if the underlying signals are already encoded. Overall, our results support the six canonical ORFs as the most effective and standardized input representation for DeepTYLCV, and they were therefore selected as the final model. At the same time, the potential context-specific roles of additional small ORFs remain an important direction for future investigation. To facilitate future studies, we made the model weights of the extended eight-ORF along with six-ORF version publicly available on our web server.

### DeepTYLCV blind predictions and experimental validation

**Figure 3:**
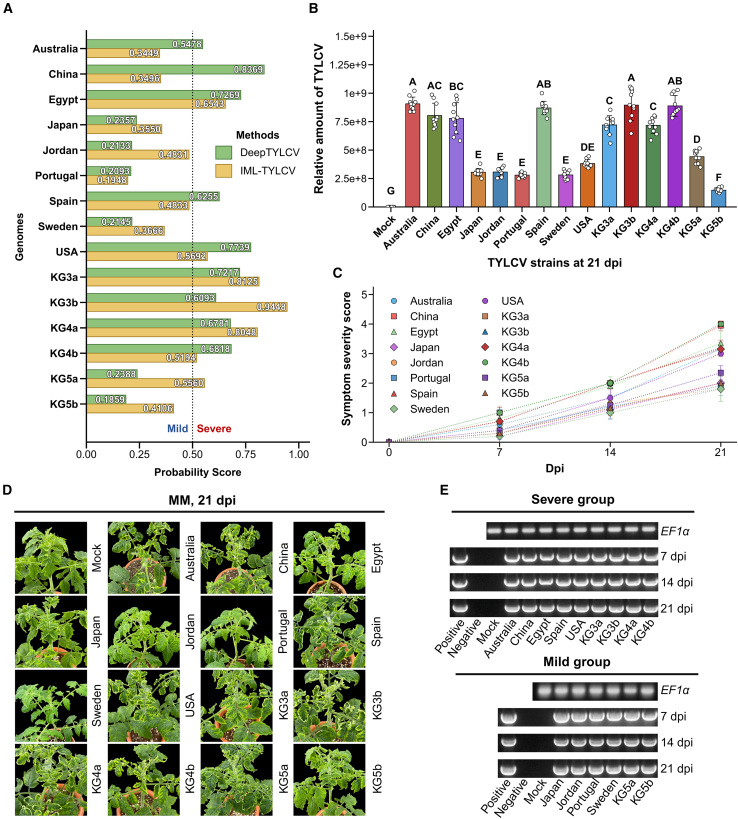
Experimental validation of DeepTYLCV predictions through symptom development and viral quantification in tomato plants. Moneymaker (MM) plants were agroinoculated with 15 TYLCV infectious clones. **(A)** Prediction probabilities generated by DeepTYLCV and IML-TYLCV for severity classification across the 15 TYLCV isolates. **(B)** Quantification of TYLCV DNA accumulation by qPCR at 21 days post-inoculation (dpi). Viral accumulations were normalized to the tomato *EF1α* gene and presented as mean ± SD (*n* = 10). One-way ANOVA followed by Tukey’s multiple comparison test was used to assess differences among isolates. Bars labeled with different uppercase letters indicate significant differences at *p* < 0.05. **(C)** Time-course analysis of symptom severity, evaluated weekly on a 0–4 scale. **(D)** Representative disease symptoms observed in inoculated MM plants at 21 dpi. **(E)** Representative PCR detection of TYLCV infection in inoculated plants using TYLCV-specific primers at 7, 14, and 21 dpi, resolved on a 1% agarose gel. Lane Positive, positive control; lane Negative, no-template control; lane Mock, total DNA from mock-inoculated plants; lanes Australia to KG5b, total DNA extracted from leaves infected with the 15 TYLCV infectious clones used in this study.

To evaluate the real-world predictive utility of DeepTYLCV, we performed blind prediction on 15 TYLCV isolates, including 9 internationally sourced reference isolates, TYLCV-Australia (KX347097), TYLCV-China (PX425771), TYLCV-Egypt (AY594174), TYLCV-Japan (LC790476), TYLCV-Jordan (EF054894), TYLCV-Portugal (AF105975), TYLCV-Spain (MH680957), TYLCV-Sweden (HF548825), and TYLCV-USA (PP505780), and six Korean field isolates collected in 2023, including TYLCV-KG3a (PP179300), TYLCV-KG3b (PP179301), TYLCV-KG4a (PP179261), TYLCV-KG4b (PP179297), TYLCV-KG5a (PP179273), and TYLCV-KG5b (PP179283). DeepTYLCV predictions were subsequently validated through symptom-based assays in tomato plants (Figure 3A).

We first constructed a set of 15 TYLCV infectious clones corresponding to these isolates. All clones were confirmed to be systemically infectious in *Nicotiana benthamiana*, as evidenced by the development of typical TYLCV-like symptoms, including upward leaf curling and interveinal yellowing (Supplemental Figures 5 and 6). In contrast, mock-inoculated plants remained asymptomatic throughout the experiment. These results confirmed that all synthesized and field-derived clones were fully competent for infection and suitable for downstream pathogenicity assays in tomato.

We next evaluated pathogenicity in the tomato (*Solanum lycopersicum*) cultivar Moneymaker. Four-week-old tomato seedlings were agroinoculated with each clone, and symptom progression was recorded at 7, 14, and 21 days post-inoculation (dpi). Disease severity was assessed using a 0–4 scale, where 0 indicated no visible symptoms; 1, mild leaf curling; 2, moderate curling with slight yellowing and reduced emergence of new leaves; 3, severe curling and deformation with clear reduction in young leaves; and 4, severe stunting with strong yellowing and extensive deformation of young leaves (Supplemental Figure 7). In parallel, viral accumulation was quantified using quantitative real-time PCR (qPCR) targeting TYLCV DNA and normalized against the endogenous *EF1α* reference gene.

Clear phenotypic differences emerged over time between isolates predicted as severe and mild. At 7 dpi, plants infected with isolates classified as severe by DeepTYLCV had already begun to exhibit early symptoms, including leaf curling and mild yellowing, whereas plants infected with mild isolates displayed only subtle phenotypic changes. By 14 dpi, the severe isolates (TYLCV-Australia, -China, -Egypt, -Spain, -USA, and all Korean isolates except KG5a and KG5b) displayed pronounced stunting, leaf deformation, and chlorosis. These symptoms became more severe by 21 dpi, with severe isolates reaching disease scores of 3–4, whereas mild isolates (TYLCV-Japan, -Jordan, -Portugal, -Sweden, -KG5a, and -KG5b) remained at scores of 2 or below (Figure 3C and 3D, Supplemental Figure 8, and Supplemental Table 2).

Quantification of viral accumulation further supported the DeepTYLCV predictions. All infected plants showed significantly higher TYLCV DNA accumulation than mock controls (*p* < 0.001; Supplemental Figure 9). At 7 dpi, viral accumulation ranged from 3.18 × 10^6^ to 5.01 × 10^7^ copies/μl. By 21 dpi, the highest viral accumulations were observed in TYLCV-Australia (9.06 × 10^8^ ± 5.87 × 10^7^), TYLCV-KG3b (8.95 × 10^8^ ± 1.34 × 10^8^), TYLCV-KG4b (8.88 × 10^8^ ± 9.17 × 10^7^), TYLCV-Spain (8.70 × 10^8^ ± 5.92 × 10^7^), TYLCV-China (8.03 × 10^8^ ± 1.08 × 10^8^), and TYLCV-Egypt (7.79 × 10^8^ ± 1.38 × 10^8^), consistent with their classification as severe isolates. In contrast, mild isolates such as TYLCV-Japan (3.05 × 10^8^ ± 3.35 × 10^7^), TYLCV-Jordan (3.07 × 10^8^ ± 3.74 × 10^7^), TYLCV-Portugal (2.80 × 10^8^ ± 1.97 × 10^7^), TYLCV-Sweden (2.81 × 10^8^ ± 3.46 × 10^7^), and TYLCV-KG5b (1.48 × 10^8^ ± 1.88 × 10^7^) showed substantially lower viral accumulation. A few isolates, including TYLCV-USA (3.82 × 10^8^ ± 3.47 × 10^7^), TYLCV-KG5a (4.42 × 10^8^ ± 5.73 × 10^7^), TYLCV-KG3a (7.20 × 10^8^ ± 8.19 × 10^7^), and TYLCV-KG4a (7.17 × 10^8^ ± 7.07 × 10^7^), showed intermediate accumulation levels. One-way ANOVA confirmed a significant effect of isolate on viral accumulation (*F*_15,144_ = 176.963, *p* < 0.001, *η*^2^ = 0.9485), and Tukey’s multiple comparisons test further categorized the isolates into seven statistically distinct viral accumulation groups (A–G; Figure 3B and Supplemental Table 3).

Importantly, both symptom severity assessment and viral accumulation measurements were in complete agreement with the blind predictions generated by DeepTYLCV (Figure 3A). Severe isolates induced strong symptoms and high viral accumulation, while mild isolates produced weaker symptoms and lower accumulation levels. PCR detection further confirmed TYLCV infection in all inoculated plants (Figure 3E). In summary, the DeepTYLCV model achieved 100% concordance (15/15) between predicted and observed severity classes. This result underscores the practical value of DeepTYLCV for predictive pathogenicity assessment and early screening of emerging TYLCV variants.

Evaluation of IML-TYLCV in the blind prediction experiment revealed that, although it performed reasonably well on Korean isolates, it lacked generalizability to isolates from other geographic regions. This limitation highlights the necessity of training on globally diverse genomes and developing integrative frameworks capable of capturing broader sequence variation. In contrast, the perfect agreement achieved by DeepTYLCV across both international and Korean isolates demonstrates its ability to identify severity-associated sequence signatures that remain robust across geographic backgrounds.

### Model interpretation analysis

To determine whether DeepTYLCV captures biologically meaningful sequence patterns associated with TYLCV severity, we analyzed the feature representations learned by the multi-scale convolutional localization (MSCL) module of the final model. Specifically, we used 1D-Grad-CAM++ (Zhang et al., 2022) to capture the importance score for each convolutional scale, followed by a gradient-weighted averaging to generate the final residue-level importance scores. These scores reflect the relative contribution of each amino acid to the model’s severity prediction. To further validate the biological relevance of identified patterns, we compared the model-highlighted regions with statistically enriched sequence motifs extracted from STREME (Bailey, 2021). The resulting motifs were mapped back to their corresponding positions in the original full-length TYLCV genomes, enabling precise localization of functionally relevant sequence segments (Yan et al., 2025).

**Figure 4:**
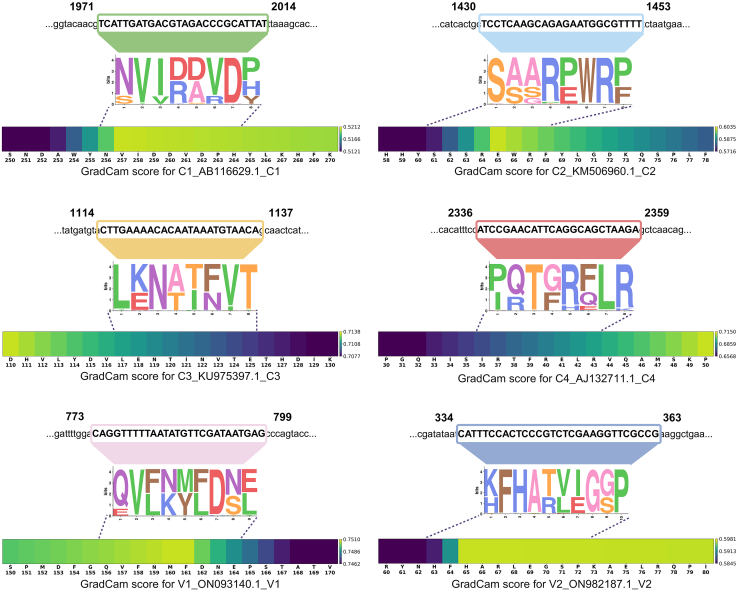
Visualization of 1D-Grad-CAM++-derived importance score and STREME-identified motifs mapped onto the original TYLCV genomes. 1D-Grad-CAM++ heatmaps and STREME motif analyses highlighting amino acid residues and corresponding nucleotide positions that contribute most strongly to DeepTYLCV predictions across six TYLCV ORFs show regions identified as important.

To evaluate whether DeepTYLCV effectively focuses on biologically relevant sequence regions, we examined representative samples, including C1_AB116629.1_C1, C2_KM506960.1_C2, C3_KU975397.1_C3, C4_AJ132711.1_C4, V1_ON093140.1_V1, and V2_ON982187.1_V2 (Figure 4). Importantly, mapping these computational hotspots back to the TYLCV genome revealed that DeepTYLCV consistently focuses on regions with known or plausible functional regions. In the complementary-sense ORFs, the model highlighted a motif in the C1 replication-associated ORF spanning positions 256–264, overlapping the highly conserved Walker B motif (257–263) (Clérot and Bernardi, 2006), which is essential for ATPase and helicase activity during replication. In the C2 transcriptional activator, the attention peak at positions 61–68 overlapped with a region where substitutions or mutations at positions 61 and 68 have been associated with reduced pathogenicity (van Wezel et al., 2002). In the C3 replication enhancer, the highlighted positions 117–124 overlapped with the 116–125 region required for interaction with C1, while the adjacent region at positions 125–134 corresponds to the binding interface for host retinoblastoma-related protein (pRBR), which facilitates viral replication (Settlage et al., 2005). In the virion-sense V2 RNA silencing suppressor, the model focused heavily on residues 63–72, which overlap with the structurally important 61–68 region required for proper active-site positioning and symptom modulation (Zhao et al., 2018; Rishishwar and Dasgupta, 2019). Overall, these overlaps demonstrate that DeepTYLCV captures sequence signals that are strongly correlated with biologically relevant functional domains rather than relying on superficial sequence patterns.

Interestingly, the model also identified distinct hotspot regions in the V1 coat protein (positions 156–164) and C4 symptom determinant (positions 36–43) that currently lack clear experimental annotation. Because DeepTYLCV successfully recovered several previously established functional regions in other ORFs, these uncharacterized hotspots may represent candidate virulence-associated elements rather than spurious signals. This possibility is noteworthy because C4 is known to act as a multi-faceted symptom determinant (Padmanabhan et al., 2022; Zhai et al., 2022), whereas the V1 coat protein is subject to intense evolutionary pressure associated with host interaction and vector transmission. These findings suggest that DeepTYLCV may help uncover novel virulence-associated sequence determinants and provide targeted candidates for future site-directed mutagenesis and functional validation. These regions may also provide useful targets for future functional studies and could ultimately inform sequence-guided strategies for TYLCV resistance monitoring and breeding.

The interpretation analysis revealed that the model consistently allocated significantly higher attention scores to amino acids within these specific motif regions compared with surrounding residues. This pattern strengthens confidence that DeepTYLCV is leveraging biologically meaningful signals, not superficial sequence patterns, to drive accurate severity predictions. These motifs are, therefore, highly probable candidates for critical determinants of virulence and host–pathogen specificity (Ito et al., 2025).

### Ablation experiment

To elucidate the contributions of individual components within the DeepTYLCV framework, we performed two sets of ablation experiments: one focusing on input feature representations and the other on architectural modules (Supplemental Figure 10). In the first ablation, we assessed the impact of integrating biologically interpretable optCCDs with the top three PLM-based embeddings. The inclusion of optCCDs resulted in consistent improved performance across both datasets. On the training set, MCC and BACC increased by 3.72% and 1.88%, respectively, while on the independent test set, gains of 3.03% in MCC and 1.02% in BACC were observed. These results demonstrate the complementary nature of two feature types. While PLMs effectively capture global contextual sequence patterns, optCCDs contribute domain-specific biochemical and physicochemical properties relevant to prediction function. The fusion of these modalities enhances feature diversity and leads to more robust and generalizable predictions.

The second ablation investigated the architectural contributions of the transformer encoder (TE) and the MSCL. Compared with the reduced configurations, the complete model incorporating both modules consistently achieved superior performance. On the training set, BACC improved by 0.53%–2.75% and MCC by 2.56%–5.85%. On the independent test set, BACC and MCC increased by 0.45%–4.97% and 1.39%–7.12%, respectively. These findings underscore the complementary roles of two modules: TE captures long-range dependencies across the viral genome, while MSCL captures salient motif-level patterns associated with virulence. Their combination enables a hierarchical representation that improves discriminative capability across diverse TYLCV strains.

Collectively, these ablation analyses validate the design of DeepTYLCV as a hybrid and biologically informed framework. The integration of interpretable domain-specific features with complementary advanced deep learning architectures enhances both predictive performance and generalizability. As a result, DeepTYLCV is well suited to real-world applications in global surveillance, genome-guided resistance breeding, and precision virology.

### Limitations and future directions

While DeepTYLCV provides a robust framework for sequence-based virulence prediction, several limitations should be acknowledged. First, the currently available TYLCV dataset is inherently imbalanced, with severe isolates substantially overrepresented compared with mild strains. This skew likely reflects agricultural sampling bias, as severe outbreaks cause greater economic damage and are therefore more likely to be sampled, sequenced, and reported in the literature. Although DeepTYLCV performed well despite this imbalance, expanding the representation of mild isolates will be important to improve dataset balance and further strengthen model robustness. Second, our experimental validation was conducted under controlled greenhouse conditions and was limited by the availability of geographically diverse isolates. Although the blind validation results strongly support the predictive utility of DeepTYLCV, broader validation across multiple environments, host backgrounds, and isolate collections will be necessary to more fully establish its generalizability under real-world conditions. Finally, while DeepTYLCV demonstrates high accuracy for TYLCV, a preliminary evaluation of 15 other monopartite begomoviruses revealed that the current model is specialized for TYLCV and does not generalize to other species without retraining (Supplemental Figure 11). Expanding this framework to a broader begomovirus population will require curation of additional species-specific training datasets. An important direction for future work will be the development of a more universal begomovirus predictor, designing an advanced computational framework that integrates the complete genome with all ORFs as a unified representation.

## Methods

### Dataset construction

To construct a comprehensive global dataset of TYLCV isolates annotated by symptom severity (mild/severe), we retrieved 1419 complete TYLCV nucleotide sequences from the NCBI GenBank database (Sayers et al., 2024) ranging from January 1, 2001, to June 22, 2024. To establish a robust, gold-standard dataset for current and future computational research, we manually verified each collected sequence by cross-referencing its corresponding literature to confirm the phenotype, and consistently reported symptoms were assigned directly to categorize its virulence as severe or mild. Conflicting reports were resolved using a majority rule. Isolates with only moderate symptoms were excluded to avoid misclassified labels. For host-dependent variation, classification was based on the primary host used in the original study. Sequences associated with patent applications (*n* = 194) and those lacking verifiable literature or clear phenotypic classification (*n* = 48) were excluded. These filtering processes resulted in a total of 1177 curated full genomes (1078 severe and 99 mild isolates) (Figure 1A). To ensure transparency and reproducibility, a comprehensive dataset summary including country of origin, host species, year of report, and GenBank accession numbers for all isolates is provided in Supplemental Tables 4 and 5. In our previous study, we found that genome-only prediction models exhibited limited transferability across diverse TYLCV isolates, highlighting the importance of incorporating proteomic information to improve generalization. Accordingly, we processed all nucleotide sequences using the NCBI ORF Finder tool to identify all ORFs (Rombel et al., 2002). These ORFs correspond to the six necessary TYLCV proteins (V1, V2, C1, C2, C3, and C4).

After generating all protein sequences, we removed identical sequences across the entire pool to ensure non-redundancy, and the remaining sequences were divided into two datasets: 80% of the data (1706 severe and 190 mild ORFs) was used for model training, while the remaining 20% (439 severe and 35 mild ORFs) was used to check model transferability. Importantly, to prevent data leakage and ensure the model’s robustness, we implemented a nucleotide sequence-level split: all ORFs originating from a single nucleotide genome were assigned either to the training dataset or entirely to the testing dataset. In our framework, during model training and independent testing, each ORF was treated as an individual prediction unit. The rationale behind this design is that each ORF contains distinct sequence characteristics and may capture mild- or severe-associated patterns from different functional perspectives while still inheriting the same virulence label from its source genome. However, although the ORF-based formulation increases the number of model inputs, the true biological unit of independence remains the genome, not the individual ORF. Therefore, this design should be interpreted as a structured decomposition of the viral genome into correlated gene-level inputs, rather than as an inflation of the effective sample size.

To enable blind prediction and subsequent experimental validation, we curated a separate set of 15 TYLCV isolates, comprising 9 representative isolates selected from well-studied international regions and 6 uncharacterized sequences obtained from a 2023 survey of Korean tomato fields. For this case-study analysis, in which genome-level virulence prediction was required in practice, we averaged the probability scores of six ORFs. This approach allowed us to convert ORF-level outputs into a single genome-level decision while still preserving the interpretability benefits of the ORF-based framework.

### Feature representation using protein language, classical natural processing language models, and conventional descriptors

To extract informative representations from TYLCV-encoded ORFs, we utilized a diverse ensemble of 12 state-of-the-art PLMs; each model was pre-trained on millions of sequences to capture contextual, structural, and evolutionary features. The PLM set included four variants of evolutionary scale modeling, ESM (Rives et al., 2021; Lin et al., 2023), ESM-1V (Meier et al., 2021), ESM-1B (Rives et al., 2021), and ESM-2, and six models from the ProtTrans (Elnaggar et al., 2021) family, PTAB, PTBB, ProtTrans-T5-BFD, PTU, ProtTrans-T5-XL-Uniref50, and ProtTrans-XLNet-Uniref100. Additionally, we incorporated representations from PLUS RNN (Min et al., 2021) and Bepler (Bepler and Berger, 2019) embeddings, as well as general-purpose sequence embeddings, including GloVe (Pennington et al., 2014), FastText (Joulin et al., 2017), and Word2Vec (Mikolov et al., 2013), which have been adapted for protein sequence inputs. Supplemental Table 6 summarizes the specific model variants used, including their versions and embedding dimensions.

To enhance model generalizability and capture complementary physicochemical and compositional information, we supplemented the PLM/NLP-based embeddings with 17 handcrafted sequence-based descriptors. These included 11 widely used descriptors: amino acid composition, dipeptide composition, grouped dipeptide composition, grouped tripeptide composition, CTD composition/CTD transition/CTD distribution descriptors, *k*-spaced amino acid pair composition, quasi-sequence order, composition of *k*-spaced amino acid group pairs, and dipeptide deviation from expected mean. We further incorporated six additional descriptors: amphiphilic pseudo amino acid composition, composition of *k-spaced amino acid pairs*, sequence-order coupling number, Moran autocorrelation, Geary autocorrelation, and general amino acid composition. These descriptors were extracted by employing the iFeatureOmega tool (Chen et al., 2022).

### Construction of the DeepTYLCV framework

The overall framework of DeepTYLCV comprises five primary modules: (1) the feature processing module extracts, projects, and stacks different high-dimensional representations into meaningful low-dimensional embeddings (Figure 1B); (2 and 3) the global–local feature learning module comprises two submodules: TE, which functions as an adapter, facilitating interactions among features from distinct PLMs/NLPs and aligning them into a shared latent space, and MSCL, which identifies local motif-pattern signatures (Figure 1C); (4) the conventional descriptor feature selection module identifies the optCCDs that capture interpretable physicochemical and composition-based features (Figure 1D); and (5) the classifier module integrates all representations to categorize ORFs as either severe or mild (Figure 1E). Finally, we deployed a user-friendly web server (Figure 1F).

#### Feature processing module

Let ${\left\{A_{i}\right\}}_{i=1}^{K}s\in R^{L\times A_{i}}$ represent the top-*K*-ranked feature matrices obtained from different PLMs/NLPs, where *L* represents the protein sequence length and $d_{A_{i}}$ denotes the embedding dimension of the feature *A*_*i*_. To maintain consistent input dimensions for the deep learning architecture, a maximum sequence *L* = 363 was established, corresponding to the longest sequence in the training dataset. During testing and inference, any sequences exceeding this token length were handled via direct truncation.

To efficiently integrate these representations without incurring computational costs, particularly as *K* increases, we define the fixed target width *d*_target_ for the final concatenated feature space. Rather than directly concatenating full-width PLM/NLP-based embeddings (which produce a high-dimensional tensor), we project each input feature to a proportionally smaller dimensional space using a lightweight linear layer. The projected features of them are given by

(Equation 1) $${\left\{A_{i}^{\text{prj}}\right\}}_{i=1}^{K}=\text{Linear}\left(A_{i}\right),A_{i}^{\text{prj}}\in R^{L\times d_{A_{i}}^{\text{prj}}}\text{,}$$

$$\text{where}\;d_{A_{i}}^{\text{prj}}=\left\{ \begin{array}{c} \lfloor \frac{d_{\text{target}}}{K}\rfloor ,for\;i\in \left\{1,2,\ldots ,K-1\right\},\\ \lfloor \frac{d_{\text{target}}}{K}\rfloor +\left(d_{\text{target}\;}\;\mathrm{mod}\;K\right),\text{for}\;i=K. \end{array}\right.$$

This allocation ensures that the total width of all project features exactly matches *d*_target_, preserving representational integrity while maintaining computational efficiency.

Specifically, after obtaining the projected features ${\left\{A_{i}^{\text{prj}}\right\}}_{i=1}^{K}$, we stacked them along the feature dimension to construct a unified representation. A learnable classification token, denoted as $\text{CLS}\in R^{1\times d_{\text{target}}}$, was used to form a global sequence-level representation. The final input sequence is defined as

(Equation 2) $$\begin{array}{c} A_{\text{stacked}}=\text{concat}\left(\text{CLS},\text{stack}\left(A_{i}^{\text{prj}}\right)\right),A_{\text{stacked}}\in R^{\left(L+1\right)\times d_{\text{target}}} \end{array}\text{.}$$

The inclusion of the classification (CLS) token was inspired by a previous study (Devlin et al., 2019), which introduced a special classification token at the start of the input sequence during pre-training. This token aggregates information across the entire sequence, enabling the model to learn a comprehensive global context.

Next, we added learnable positional embeddings ${PE}_{\text{stacked}}\in R^{\left(L+1\right)\times d_{\text{target}}}$ to retain order information, which was fed into the input of the subsequent module:

(Equation 3) $$\begin{array}{c} H^{\left(0\right)}=A_{\text{stacked}}+{PE}_{\text{stacked}} \end{array}\text{.}$$

#### Global–local feature learning module

The processed features from the previous module, *H*^(0)^, are then passed through *L*_enc_ layers of TE, each defined recursively as

(Equation 4) $$\begin{array}{c} H^{\left(l\right)}={\text{TransformerLayer}}^{l}\left(H^{\left(l-1\right)}\right),\forall l\in \left\{1,2,\ldots ,L_{\text{enc}}\right\} \end{array}\text{.}$$

Each transformer layer (Vaswani et al., 2017) consists of a multi-head self-attention (MHSA) mechanism followed by a feedforward network (FFN), both wrapped with residual connections and layer normalization (LN):

(Equation 5) $$\begin{array}{l} \overset{˜}{H^{\left(l\right)}}=\text{LN}\left(H^{\left(l-1\right)}+\text{MHSA}\left(H^{l-1}\right)\right)\\ H^{\left(l\right)}=\text{LN}\left(\overset{˜}{H^{\left(l\right)}}+\text{FFN}\left(\overset{˜}{H^{\left(l\right)}}\right)\right) \end{array}\text{.}$$

Let ${\tilde{A}}_{\text{stacked}}=H^{\left(L_{\text{enc}}\right)}$ denote the output of the final layer. From this, we split it into CLS token embeddings and sequence embeddings, denoted as $F_{\text{global}}\in R^{{1\times d}_{\text{target}}}$ and ${\tilde{A}}_{\text{seq}}\in R^{{L\times d}_{\text{target}}}$, respectively. ${\tilde{A}}_{\text{seq}}$ is then passed to the MSCL for local feature refinement.

To capture local sequence motifs relevant to TYLCV protein function, we used 1D convolutional (Conv1D) layers (Yamashita et al., 2018), which are well suited to identifying biologically meaningful motifs by applying filters to adjacent sequence elements. We employed three-scale convolutional blocks, each with a different kernel size *k*_*i*_, to capture motifs at multiple spatial scales. Each block comprises two consecutive Conv1D layers. The encoded sequence features ${\tilde{A}}_{\text{seq}}^{T}$ (transposed to match Conv1D input format) are processed as follows:

(Equation 6) $$\begin{array}{l} B_{i}=\sigma \left(W_{1}^{\left(i\right)}⊛{\tilde{A}}_{\text{seq}}^{T}+b_{1}^{\left(i\right)}\right),B_{i}\in R^{\frac{d_{\text{target}}}{2}\times L_{\text{out}}^{\left(1\right)}}\\ B_{i}^{\prime }=\sigma \left(W_{2}^{\left(i\right)}⊛B_{i}+b_{2}^{\left(i\right)}\right),{\;B}_{i}^{\prime }\in R^{d_{\text{target}}\times L_{\text{out}}^{\left(2\right)}} \end{array}\text{.}$$

Here, ⊛ represents the Conv1D operation, and $W_{1}^{\left(i\right)}\text{and}\;W_{2}^{\left(i\right)}$ are learnable convolutional filters with dimensions $R^{\frac{d_{\text{target}}}{2}\times d_{\text{target}}\times k_{i}}$ and $R^{d_{\text{target}}\times \frac{d_{\text{target}}}{2}\times k_{i}}$, respectively. The terms $b_{1}^{\left(i\right)}$ and $b_{2}^{\left(i\right)}$ are bias terms, and *σ* is the ReLU activation function. Padding $p=\frac{k_{i}}{2}$, stride *s*, and dilation *d* are configured to preserve the same length as the original sequence.

The output sequence lengths after two convolutional layers ($L_{\text{out}}^{\left(1\right)}$ and $L_{\text{out}}^{\left(2\right)}$) are computed as

(Equation 7) $$\begin{array}{l} L_{\text{out}}^{\left(1\right)}=\lfloor \frac{L+2p-d\left(k_{i}-1\right)-1}{s}+1\rfloor \text{,}\\ L_{\text{out}}^{\left(2\right)}=\lfloor \frac{L_{\text{out}}^{\left(1\right)}+2p-d\left(k_{i}-1\right)-1}{s}+1\rfloor \end{array}\text{.}$$

Next, we apply average pooling to aggregate sequence information, transforming the 2D feature maps into 1D vectors suitable for the fully connected layers in the classifier module. This operation is formulated as follows:

(Equation 8) $$\begin{array}{c} {\bar{B}}_{i}=\frac{1}{L_{out}^{\left(2\right)}}\sum_{j=1}^{L_{out}^{\left(2\right)}}B_{i}^{\prime }\left(j\right),{\bar{B}}_{i}\in R^{d_{target}}\text{.} \end{array}$$

The pooled output from all three scales are then aggregated via bit-wise summation to form a unified local representation:

(Equation 9) $$\begin{array}{c} {{\bar{B}}_{i}}^{\prime }=\sum_{i=1}^{3}{\bar{B}}_{i},{{\bar{B}}_{i}}^{\prime }\in R^{d_{target}} \end{array}\text{.}$$

As the feature representations from different scales will have different value ranges and distributions, the resulting representations are heterogeneous. Therefore, we apply batch normalization (BN) to stabilize the final feature representation:

(Equation 10) $$\begin{array}{c} F_{\text{local}\;}=\text{BN}\left({{\bar{B}}_{i}}^{\prime }\right),F_{\text{local}\;}\in R^{d_{\text{target}}} \end{array}\text{.}$$

#### Conventional descriptor feature selection module

After obtaining 17 different conventional descriptors, we constructed a single high-dimensional feature vector by linearly concatenating all these descriptors, referred to as CCDs, denoted as *F*_CCDs_. Although these descriptors capture diverse biological and physicochemical properties, their high dimension can lead to overfitting and increased computational load. To address this issue, we employed LightGBM-based (Ke et al., 2017) feature importance analysis to rank and select informative features. Features with non-zero importance score were retained. Subsequently, to eliminate differences in magnitude among the diverse features, we standardized these selected features to a mean of zero and unit variance using the StandardScaler module from the scikit-learn library (Pedregosa et al., 2011). This process produced the final, standardized optimal descriptor vector $F_{\text{optCCDs}}\in R^{d_{\text{optCCDs}}}$. This representation was integrated with PLM/NLP-based embeddings prior to classification in the following module.

#### Classifier module

In the final stage of the framework, we integrated the global feature *F*_global_ (from the TE) and local feature *F*_local_ (from the MSCL) via residual connection and deliberately adopted a late-fusion strategy at this stage, concatenating this combined deep representation with the optimal conventional descriptors *F*_optCCDs_. The established findings in prior study experiments that incorporated fusion at earlier stages of the pipeline hindered the learning of informative representations due to the difficulty of capturing high-level features within a prematurely shared joint representation (Stahlschmidt et al., 2022). This late-fusion design ensures that the model first learns rich contextual representations from the PLM/NLP-based embeddings while allowing the conventional descriptors to contribute complementary information directly at the classification boundary. This forms the final input feature:

(Equation 11) $$\begin{array}{c} F_{\text{final}}=\text{concat}\left(F_{\text{global}}+F_{\text{local}},F_{\text{optCCDs}}\right),F_{\text{final}}\in R^{d_{\text{target}}+d_{\text{optCCDs}}} \end{array}\text{.}$$

The unified representation *F*_final_ was passed through the FFN, yielding the final prediction logits for binary classification (mild or severe ORFs):

(Equation 12) $$\begin{array}{c} C_{\text{out}}=\text{FFN}\left(F_{\text{final}}\right),C_{\text{out}}\in R^{2}\text{.} \end{array}$$

### Optimization process

#### Loss function

To address the class imbalance during training, we adopted focal loss (FL) (Lin et al., 2017), which modulates the loss contribution of each class, thereby assigning greater significance to minority class samples without overemphasizing the majority class. The FL is defined as follows:

(Equation 13) $$\begin{array}{c} L=FL\left(P_{t}\right)=-\alpha_{t}{\left(1-P_{t}\right)}^{\gamma }\cdot \log\left(P_{t}\right)\text{,} \end{array}$$

where *P*_*t*_ = *y*·*p* + (1 − *y*)·(1 − *p*).

Here, *α*_t_ is the class-specific weighting factor, and *γ* is the focusing parameter that controls the rate at which samples are downweighted.

#### Model optimization and evaluation metrics

To maximize the performance of the DeepTYLCV model, we performed an extensive hyperparameter optimization combined with five-fold cross-validation to ensure reliable train and validation. The model weights were optimized by employing AdamW (Loshchilov, 2017), and a learning rate scheduler was employed to gradually decrease the learning rate over epochs, which improved convergence stability. The detailed information of the hyperparameter search space is provided in Supplemental Table 7.

Furthermore, we utilized commonly used evaluation metrics to evaluate model performance (Sangaraju et al., 2024; Tran et al., 2025b; Nguyen et al., 2025), including BACC, sensitivity, specificity, F1, MCC, and AUC. Since the dataset is highly imbalanced, we chose BACC as our primary metric for model selection and comparison.

### Construction of infectious clones and plant assays

Nine representative TYLCV sequences originating from Australia, China, Egypt, Japan, Jordan, Portugal, Spain, Sweden, and the United States were synthesized (Macrogen, Korea), and we cloned them into the binary vector pCAMBIA1303 using *Kpn*I and *Hin*dIII (Takara, Japan). Infectious clones of six Korean TYLCV isolates (collected in 2023) were constructed by amplifying IC1 and IC2 fragments, cloning them into the pJET vector (Thermo Fisher Scientific, USA), and assembling into the pCAMBIA1303 (1.1-mer) via a three-piece ligation using *Bam*HI, *Sph*I, and *Hin*dIII (Takara) (Kil et al., 2014). All recombinant plasmids were introduced into *Agrobacterium tumefaciens* GV3101 by the freeze–thaw method.

Moneymaker was used in all inoculation experiments. Plants were cultivated in a walk-in growth chamber at Sungkyunkwan University under controlled conditions (16 h light/8 h dark at 28°C/22°C day/night). Four-week-old plants were agroinoculated using needle and pipettes (Vo et al., 2022). For each TYLCV clone, 10 plants (5 plants per replicate) were used, with mock-inoculated plants as negative controls. *A. tumefaciens* carrying the recombinant plasmid was cultured at 28°C in LB medium containing rifampicin, gentamicin, and kanamycin until OD_600_ 1.0.

### Symptom evaluation and viral quantification

We monitored inoculated plants weekly for symptom development and recorded symptom severity score at 7, 14, and 21 dpi. Modified symptom severity score was assessed on a 0–4 scale (Friedmann et al., 1998). For viral detection, we collected leaf samples weekly and extracted total DNA using the FavorPrep Plant Genomic DNA Extraction Kit (Favorgen, Taiwan), then PCR detection was analyzed using TYLCV-det-F/R in a total volume of 20 μl containing 1 μl diluted DNA (100 ng/μl), 1 μl primers (10 μM), and 10 μl Phusion Plus PCR Master Mixes (Thermo Fisher Scientific) under standard PCR conditions for each plant every week after inoculation.

We quantified TYLCV DNA levels for viral accumulation by qPCR using a reaction of SYBR pre-mix Ex Taq (Takara) with primers specific to the V1 gene in a total volume of 10 μl containing 1 μl diluted DNA (50 ng/μl), 1 μl primers (10 μM), and 5 μl SYBR pre-mix Ex Taq Master Mix and normalized the result using the elongation factor 1α (*EF1α*) gene as the internal control. The qPCR was performed in a Rotor Gene Q thermocycler (QIAGEN, Germany). Each reaction was performed in triplicate, and relative viral accumulation was calculated using the 2^−ΔΔCT^ method (Livak and Schmittgen, 2001). The primer information is provided in Supplemental Table 8.

### Statistical analysis

Plant assays were repeated twice (*n* = 10) for each treatment, and all experiments were performed with three biological replicates for viral DNA analysis. Symptom severity scores and viral accumulation are presented as mean ± SD at 7, 14, and 21 dpi. Statistical differences for viral accumulation among TYLCV isolates were evaluated using one-way ANOVA, with a statistical significance threshold of *p* < 0.05and reported effect sizes (partial η^2^).

## Data and code availability

The DeepTYLCV scripts are available on GitHub (https://github.com/cbbl-skku-org/DeepTYLCV/). The training and independent test sets used in this study are available from https://github.com/cbbl-skku-org/DeepTYLCV/tree/main/data/. Experimental data are available from the authors upon reasonable request.

## Funding

This work was supported by the Korea Institute of Planning and Evaluation for Technology in Food, Agriculture and Forestry (IPET) through the Cutting-Edge Precision Breeding Development Program, funded by the Ministry of Agriculture, Food and Rural Affairs (MAFRA) (RS-2025-02216897). This work was carried out with the support of the “Cooperative Research Program for Agriculture Science and Technology Development (Project No. RS-2025-02273065),” Rural Development Administration, Republic of Korea; the 10.13039/501100003725National Research Foundation of Korea (NRF), funded by the 10.13039/501100014188Ministry of Science and ICT, Republic of Korea (grant no. RS-2024-00344752); the Department of Integrative Biotechnology, Sungkyunkwan University (SKKU), and the BK21 FOUR Project, Republic of Korea.

## Acknowledgments

We would like to thank the Korea Bio Data Station (K-BDS) for providing computing resources and technical support (KBDSC-2025-KBDS-0153). We also appreciate the assistance and encouragement from members of CELTECH and CBBL. No conflict of interest is declared.

## Author contributions

S.L. and B.M. conceived the project and designed the experiments. N.B. performed all experiments related to TYLCV. H.S. and D.T.T. performed bioinformatics and machine learning studies. V.K.S. performed web server development. H.I. and M.K. contributed to wet-lab experimental preparation and literature collection. N.B., B.M., and S.L. wrote the manuscript. All authors read and approved the final manuscript.

## Supplemental information

Document S1. Figures S1–S11 and Tables S1–S8

Document S2. Article plus supplemental information
