# Supplementary material for: DeepTYLCV: An interpretable and experimentally validated AI model for predicting virulence of different tomato yellow leaf curl virus strains
Source: Plant Commun. 2026 Apr 30;7(5):101877. doi: 10.1016/j.xplc.2026.101877 (PMC13174252; doi:10.1016/j.xplc.2026.101877)
Supplement: Document S1. Figures S1–S11 and Tables S1–S8 [file mmc1.pdf]

Plant Communications, Volume *7*

Supplemental information

DeepTYLCV: An interpretable and experimentally validated AI model for predicting virulence of different tomato yellow leaf curl virus strains

Nattanong Bupi, Hariharan Sangaraju, Duong Thanh Tran, Vinoth Kumar Sangaraju, Hyojin Im, Minkwan Kim, Sukchan Lee, and Balachandran Manavalan

Supplemental information

DeepTYLCV: An interpretable and experimentally validated AI model for predicting virulence of different tomato yellow leaf curl virus strains

Nattanong Bupi[†,1], Hariharan Sangaraju[†,2], Duong Thanh Tran[†,2], Vinoth Kumar Sangaraju[2], Hyojin Im[1], Minkwan Kim[1], Sukchan Lee[*,1] and Balachandran Manavalan[*,2]

[1]Celtech Laboratory, Department of Integrative Biotechnology, College of Biotechnology and Bioengineering, Sungkyunkwan University, Suwon 16419, Gyeonggi-do, Republic of Korea.

[2]Computational Biology and Bioinformatics Laboratory, Department of Integrative Biotechnology, College of Biotechnology and Bioengineering, Sungkyunkwan University, Suwon 16419, Gyeonggi-do, Republic of Korea.

[†]Nattanong Bupi, Hariharan Sangaraju, and Duong Thanh Tran have contributed equally to this work.

*Correspondence: Sukchan Lee (cell4u@skku.edu) and Balachandran Manavalan (bala2022@skku.edu)

Abbreviations used in figures

The following table provides full definitions for all abbreviations used in the figures in the main article.

Abbreviation	Full description
AAC	Amino acid composition
APAAC	Amphiphilic pseudo amino acid composition
AUC	Area under the receiver operating characteristic curve
BACC	Balanced accuracy
CKSAAGP	Composition of k-spaced amino acid group pairs
CKSAAP	Composition of k-spaced amino acid pairs
CLS	Classification
CNN	Convolutional neural network
CTDC	CTD (Composition/Transition/Distribution) composition
CTDD	CTD distribution
CTDT	CTD transition
DDE	Dipeptide deviation from expected mean
Dpi	Day post-inoculation
ESB	ESM-1B
ESM	ESM-1
ESM2	ESM-2
ESV	ESM-1V
F1	F1 score
FFN	Feed forward network
GAAC	Grouped amino acid composition
GDPC	Grouped dipeptide composition
Geary	Geary autocorrelation
GTPC	Grouped tripeptide composition
MCC	Matthews correlation coefficient
MLP	Multi-layer perceptron
MM	Tomato cv. Moneymarker
Moran	Moran autocorrelation
NLP	Natural language processing

optCCDs	Optimal concatenated conventional descriptors
ORF	Open reading frame
PLM	Protein language model
PRNN	PLUS RNN
PTAB	ProtTrans-ALBERT-BFD
PTB	ProtTrans-T5-BFD
PTBB	ProtTrans-BERT-BFD
PTU	ProtTrans-T5-UniRef50
PTXLU	ProtTrans-XLNet-Uniref100
PTXU	ProtTrans-T5-XL-Uniref50
QSOrder	Quasi-sequence order
SEN	Sensitivity
SOCNumber	Sequence-order coupling number
SPE	Specificity

Supplemental Table 1. Error rate comparison of six ORFs in the independent test set.

ORF	Total samples	Misclassified	Error Rate
C1	133	6	4.5%
C2	71	7	9.9%
C3	87	13	14.9%
C4	62	1	1.6%
V1	74	28	37.8%
V2	47	9	19.1%

Supplemental Table 2. TYLCV symptom severity score in this study at days post-inoculation (dpi).

Genotype	Symptoms score		
	**7 dpi**	**14 dpi**	**21 dpi**
TYLCV-Australia	0.6±0.52	1.5±0.53	3.15±0.24
TYLCV-China	1±0	2±0	3.95±0.16
TYLCV-Egypt	0.8±0.42	1.9±0.31	3.35±0.41
TYLCV-Japan	0.2±0.42	1.1±0.32	2±0
TYLCV-Jordan	0.3±0.48	1.3±0.48	1.8±0.42
TYLCV-Portugal	0.4±0.52	1.2±0.42	1.9±0.32
TYLCV-Spain	0.7±0.48	2±0	3.2±0.26
TYLCV-Sweden	0.2±0.42	1±0	1.8±0.42
TYLCV-USA	0.4±0.52	1.5±0.52	3±0
TYLCV-KG3a	1±0	2±0	4±0
TYLCV-KG3b	1±0	2±0	4±0
TYLCV-KG4a	0.7±0.48	2±0	3.15±0.24
TYLCV-KG4b	1.0±0	2±0	4±0
TYLCV-KG5a	0.3±0.48	1.25±0.26	2.35±0.24
TYLCV-KG5b	0.3±0.48	1.5±0.24	2±0

Supplemental Table 3. TYLCV accumulation in this study at days post-inoculation (dpi).

Genotype	Viral accumulation (mean ± SD)		
	7 dpi	14 dpi	21 dpi
TYLCV-Australia	1.75E+07±3.39E+06	6.92E+08±7.73E+07	9.06E+08±5.87E+07
TYLCV-China	1.23E+07±3.11E+06	7.01E+08±5.83E+07	8.03E+08±1.08E+08
TYLCV-Egypt	1.24E+07±1.87E+06	6.50E+08±1.17E+08	7.79E+08±1.38E+08
TYLCV-Japan	1.75E+07±4.59E+06	2.45E+08±2.35E+07	3.05E+08±3.35E+07
TYLCV-Jordan	1.75E+07±2.55E+06	2.22E+08±1.85E+07	3.07E+08±3.74E+07
TYLCV-Portugal	1.75E+07±3.23E+06	2.02E+08±2.11E+07	2.80E+08±1.97E+07
TYLCV-Spain	2.80E+07±6.84E+06	8.89E+08±7.32E+07	8.70E+08±5.92E+07
TYLCV-Sweden	1.56E+07±1.56E+06	2.52E+08±3.81E+07	2.81E+08±3.46E+07
TYLCV-USA	1.38E+07±2.56E+06	4.64E+08±3.50E+07	3.82E+08±3.47E+07
TYLCV-KG3a	3.33E+07±6.13E+06	5.14E+08±4.00E+07	7.20E+08±8.19E+07
TYLCV-KG3b	5.01E+07±1.83E+07	1.19E+09±1.56E+08	8.95E+08±1.34E+08
TYLCV-KG4a	1.67E+07±4.88E+06	5.56E+08±4.28E+07	7.17E+08±7.07E+07
TYLCV-KG4b	4.37E+07±8.06E+06	1.13E+09±9.15E+07	8.88E+08±9.17E+07
TYLCV-KG5a	1.74E+07±2.33E+06	4.71E+08±6.67E+07	4.42E+08±5.73E+07
TYLCV-KG5b	3.18E+06±9.17E+05	1.41E+08±1.41E+07	1.48E+08±1.88E+07

Supplemental Table 4. Summary of collected TYLCV isolates for model development. (The complete dataset is provided in a separate Supplementary file.)

Supplemental Table 5. Distribution of collected TYLCV genomes by country, host species, and year of collection. (The complete dataset is provided in a separate Supplementary file.)

Supplemental Table 6. Variants and Dimensional Details of the Protein Language Models and NLP Embeddings Used.

Model	Variant	Dimension
Bepler	-	121-D
Word2Vec	-	512-D
FastText	-	512-D
GloVe	-	512-D
PLUSRNN	-	1024-D
ESM1	esm1_t34_670M_UR50S	1280-D
ESM-1v	esm1v_t33_650M_UR90S	1280-D
ESM-1b	esm1b_t33_650M_UR50S	1280-D
ESM2	esm2_t33_650M_UR50D	1280-D
ProtTransAlbertBFD	prottrans_albert_bfd	4096-D
ProtTransBertTBFD	prottrans_bert_bfd	1024-D
ProtTransXLNetUniref100	prottrans_xlnet_uniref100	1024-D
ProtTransT5BFD	prottrans_t5_bfd	1024-D
ProtTransT5XLU50	Prottrans_t5_xl_u50	1024-D
ProtTransT5UniRef50	prottrans_t5_uniref50	1024-D

Supplemental Table 7. Ranges of the Tuned Hyperparameters.

Network	Hyperparameter	Search Range
Trainer	Epochs	100
	Learning Rate	0.0001
	Batch Size	16
Transformer Encoder (TE)	Number of Multi-head SA (h_{enc})	4,8
	Transformer layers (L_{enc})	6,8
	d_{target}	128,256,512
Multi-scale convolution localization (MSCL)	Kernel Size (k_i)	[1,3,5] [5,7,9]
	Dilation (d)	1,3
	Stride (s)	1,3
Classifier	Normalization	Batch
	Dropout Layer	0.1, 0.3
	FC Layer Channels	128,64,32
Focal Loss	Alpha α_t(neg, pos)	[0.9,0.1]
	Gamma γ	2,3

Supplemental Table 8. Primer sets used for the detection of viral DNA infecting tomato and amplification of the TYLCV infectious clone in this study.

Primer name	Nucleotide sequence (5'-3')	Tm (℃)	Target size (bp)
Detection PCR			
TYLCV-det-F	ACTATGTCGAAGCGACCAGGCG	64	781
TYLCV-det-R	ATTAATTTGATATTGAATCATAGAAATAGATGCG	56	
Real-time PCR			
qPCR-TYLCV-V1-F	CTCTGGAATGAAGGAACAGGC	60	141
qPCR-TYLCV-V1-R	GAGGCATGCGTACATGCCATATAC	60	
EF1α-F	ATTGGAAACGGATATGCCCCT	62.5	188
EF1α-R	TCCTTACCTGAACGCCTGTCA	62.8	
TYLCV infectious clone			
IC1KG3-F	GGTACCGTTGAAATGAATCGGTGTCCC		1186
IC1KG3-R	AGAGGCATGCGTACATGCCATATACAGTAAC		
IC2KG3-F	GTACGCATGCCTCTAATCCAGTGTATGCAAC		1920
IC2KG3-R	GGATCCATTGCAAGACAAAAAACTTGGGG		
IC1KG4-F	GGTACCATTTACAAATATGCCCTTGTACC		1186
IC1KG4-R	CGAGGCATGCGTACATGCCATATACAGTAAC		
IC2KG4-F	GTACGCATGCCTCGAATCCAGTGTATGCAAC		1906
IC2KG4-R	GGATCCATTGCAAGACAAAAAACTTGGGG		
IC1KG5-F	GGTACCATGGTCAATGAGTACCGATTGAC		1204
IC1KG5-R	CGAGGCATGCGTACATGCCATATACAATAAC		
IC2KG5-F	GTACGCATGCCTCGAATCCAGTGTATGCAAC		1938
IC2KG5-R	GGATCCATTGCAAGACAAAAAACTTGGGG		

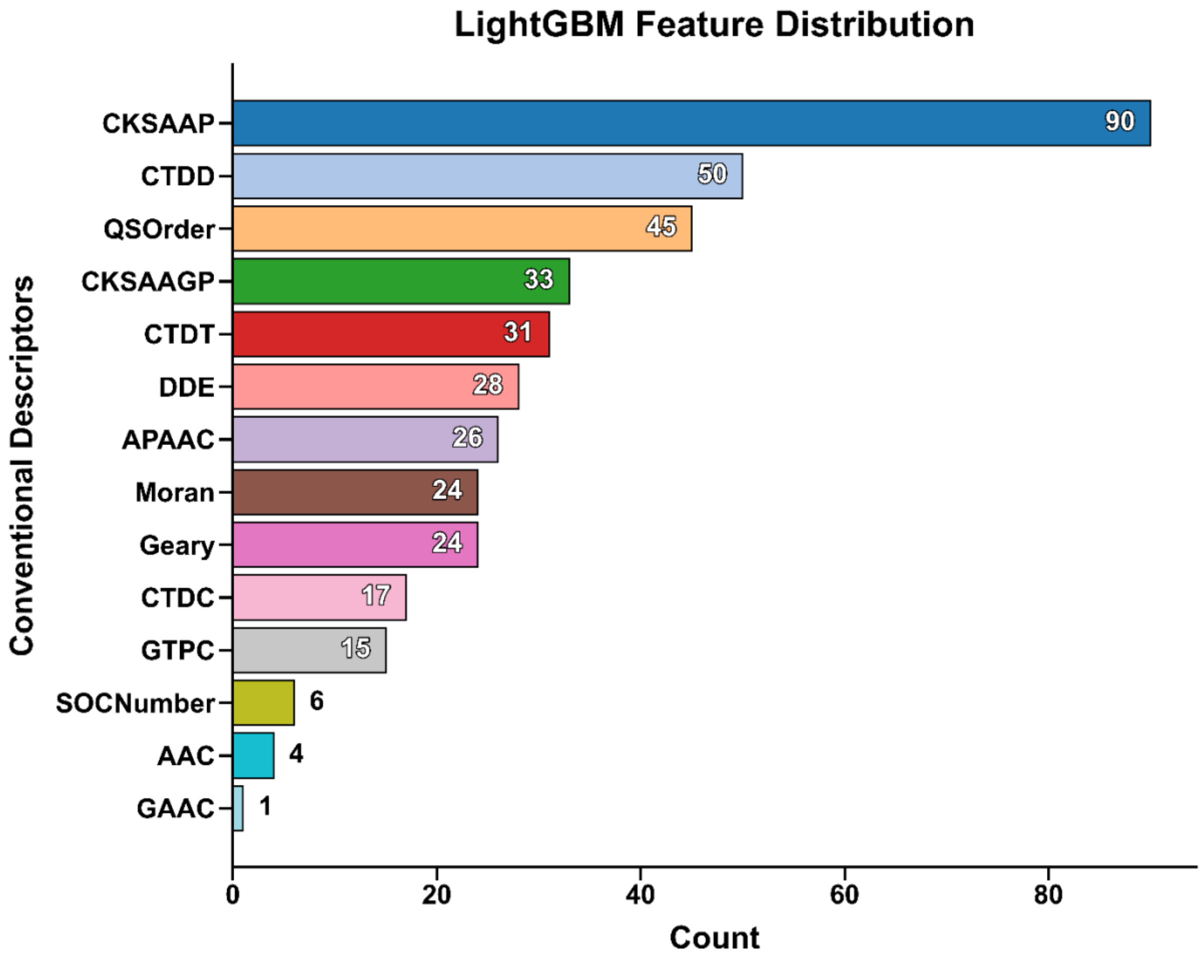

Supplemental Figure 1. Distribution of the 394 optCCDs features selected by LightGBM from 17 conventional descriptors. The x-axis (count) indicates the number of selected features from each respective conventional descriptor (y-axis).

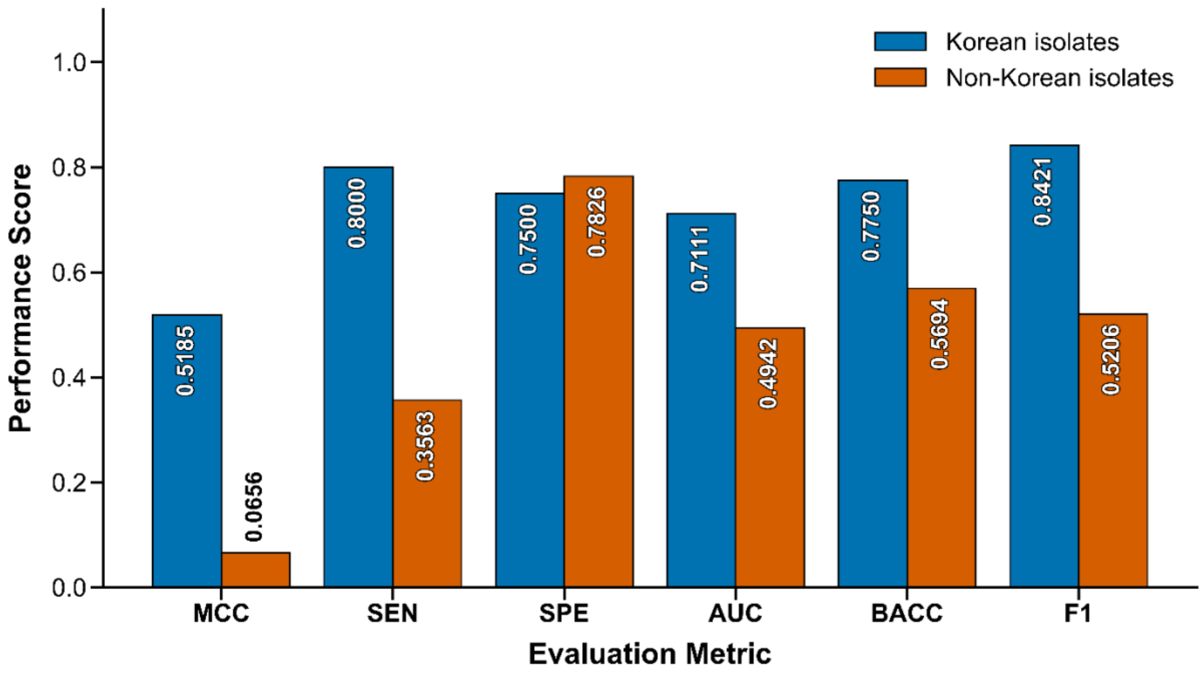

Supplemental Figure 2. Performance evaluation of the IML-TYLCV model with Korean and non-Korean isolates on an independent test set. Blue indicates the performance of Korean isolates, and orange indicates the performance of non-Korean isolates.

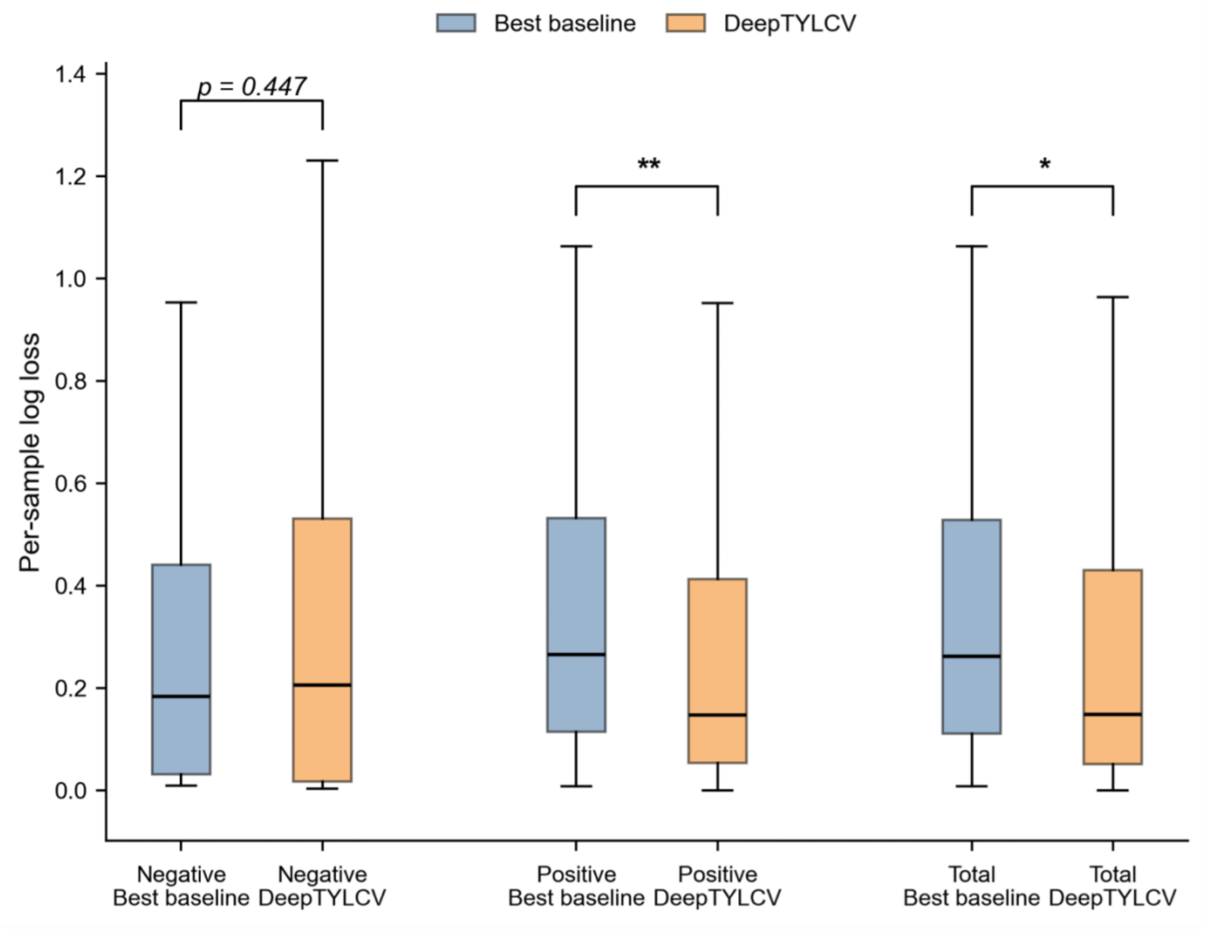

Supplemental Figure 3. Statistical comparison of per-sample log loss between the best baseline (PTAB) and DeepTYLCV models. Box plots illustrate the distribution of log loss across the negative (mild), positive (severe), and complete test datasets. Statistical significance was evaluated using paired bootstrap tests ($n = 10{,}000$ resamples). DeepTYLCV demonstrated a significant reduction in log loss on the positive set (**$p < 0.001$) and the complete independent test set (*$p < 0.05$) compared to the PTAB model. The difference in the negative set was not statistically significant ($p = 0.447$).

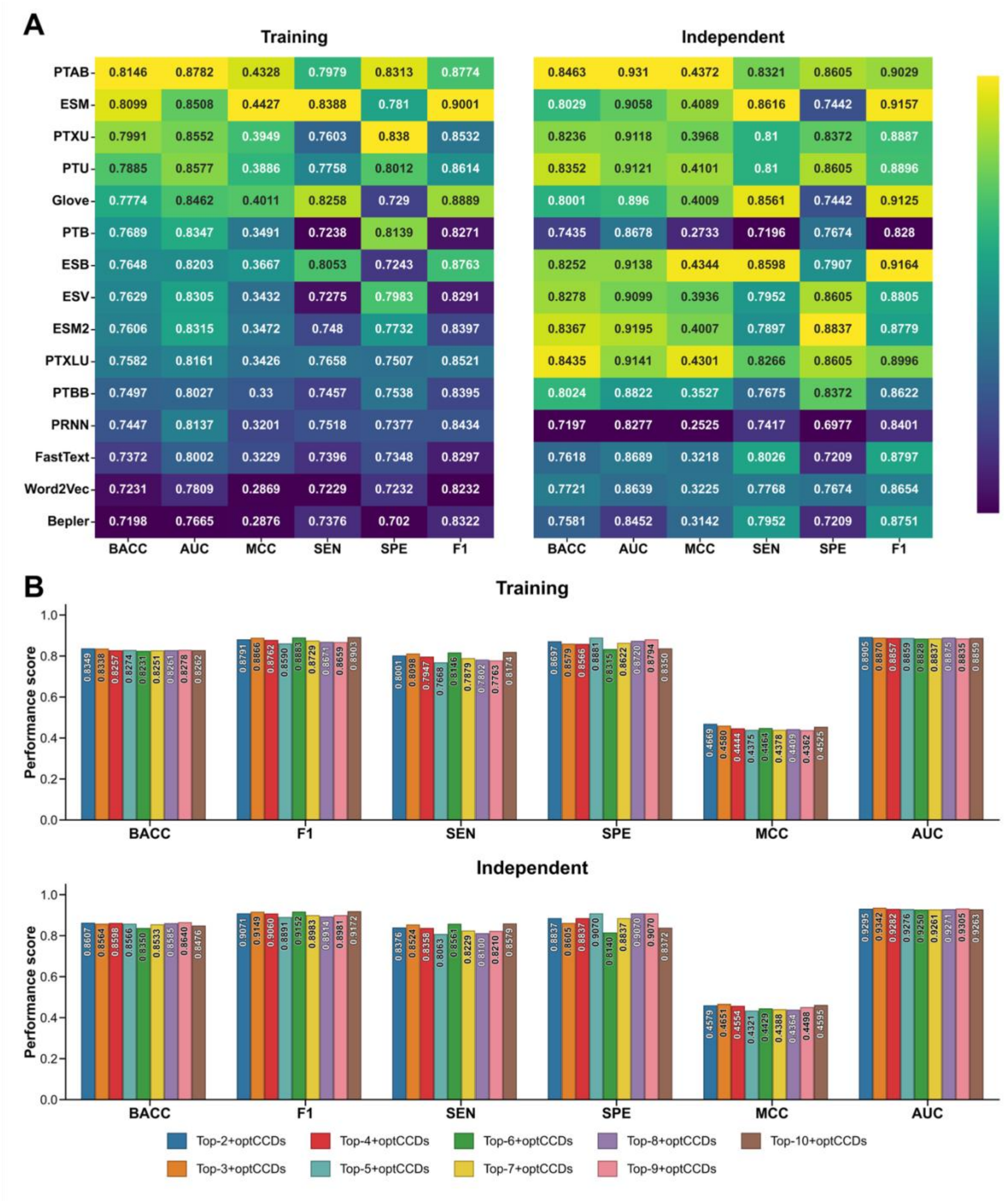

Supplemental Figure 4. Performance evaluation of an eight-ORF input-based model. (A) Performance of individual PLM/NLP-based embeddings on training and independent test sets based on eight-ORF input. (B) Performance of hybrid feature models integrating PLM/NLP embeddings with optCCDs selected via LightGBM, evaluated on both training and independent test sets.

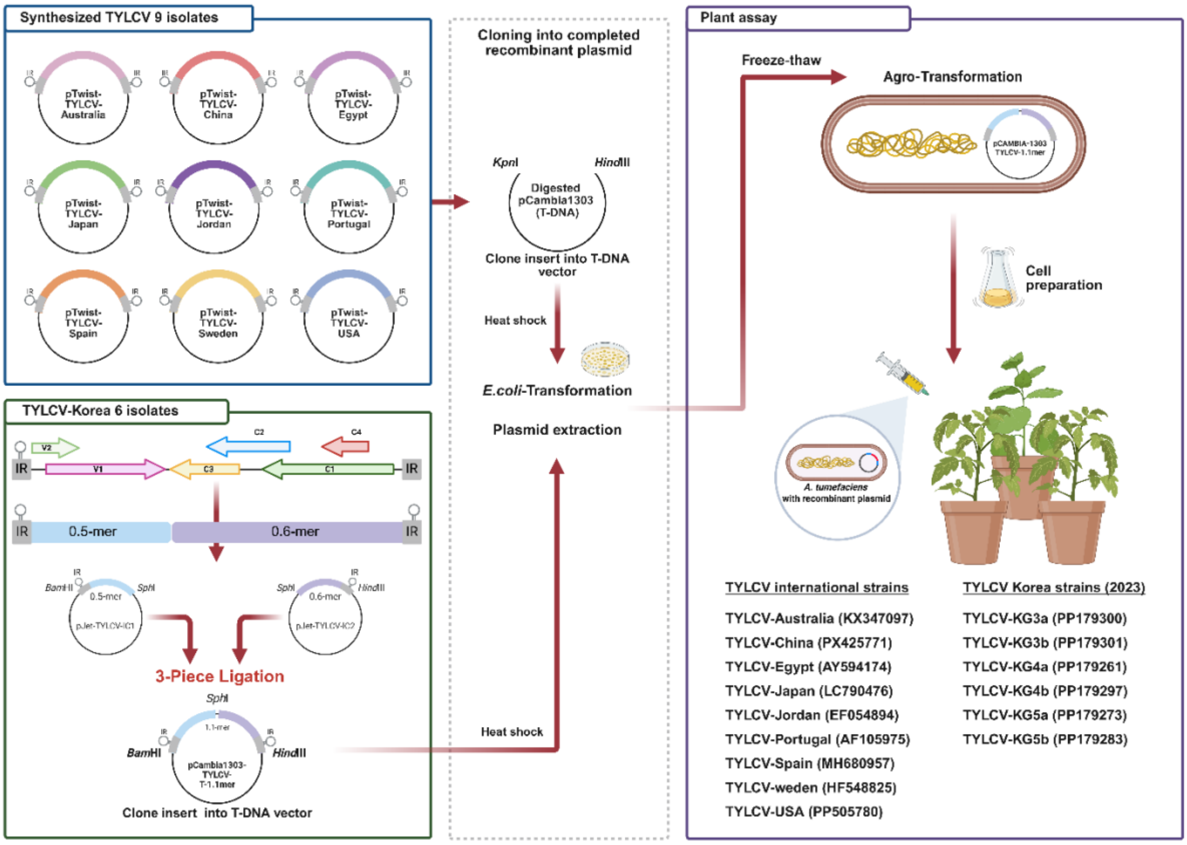

Supplemental Figure 5. Workflow for the construction of infectious clones of *Tomato yellow leaf curl virus* (TYLCV) isolates and plant assays. Nine representatives international TYLCV sequences were synthesized and cloned into pCAMBIA1303 using *Kpn*I and *Hind*III, while six Korean isolates collected in 2023 were cloned into pJET vectors and assembled into pCAMBIA1303 (1.1-mer) by three-piece ligation (*Bam*HI, *Sph*I, *Hind*III). Recombinant plasmids were transformed into *Escherichia coli* for propagation and subsequently into *A. tumefaciens* GV3101 by the freeze–thaw method. Agroinoculation was performed on MM, with mock plants used as negative controls.

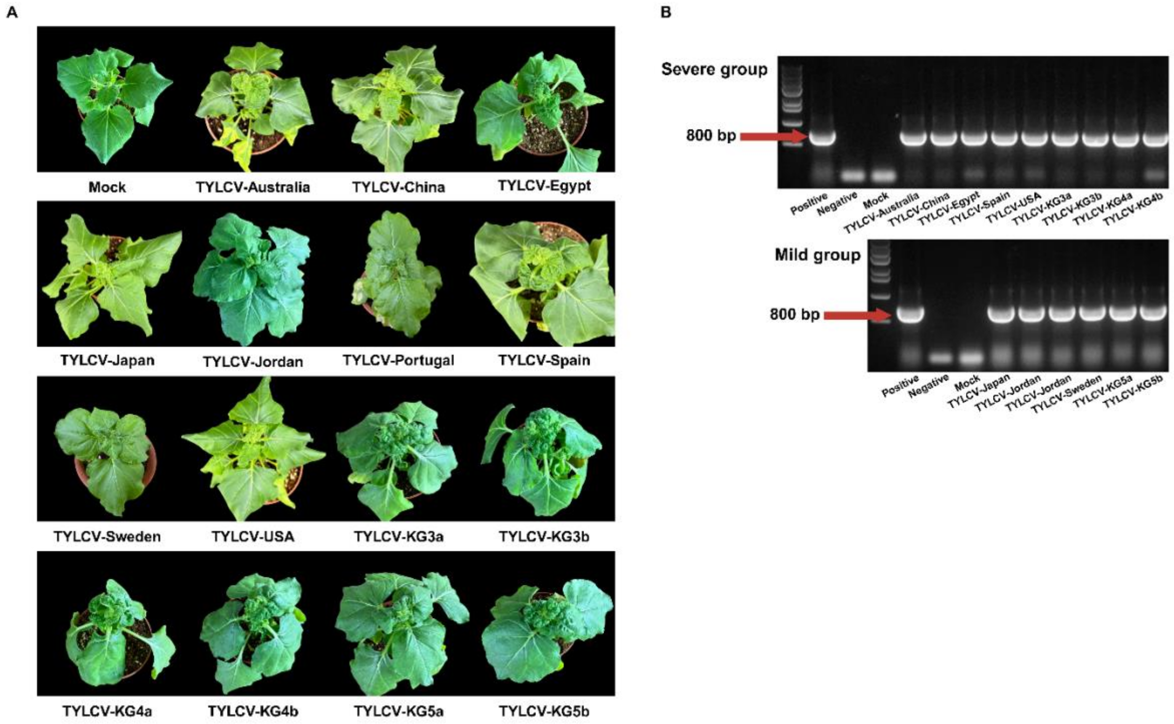

Supplemental Figure 6. Validation of TYLCV infectious clones in *N. benthamiana*. Representative symptoms of *N. benthamiana* plants at 14 days post-inoculation (dpi) with 15 TYLCV infectious clones compared to a mock-inoculated control. (A) All international clones induced upward leaf curling and yellowing. Similarly, six Korean isolates collected in 2023 produced typically symptoms of infecting begomoviruses. Mock-inoculated plants showed no visible symptoms. These results confirm that all synthesized international sequences and field-derived Korean clones are fully infectious in *N. benthamiana.* (B) The infectivity was confirmed by PCR using TYLCV detection primers.

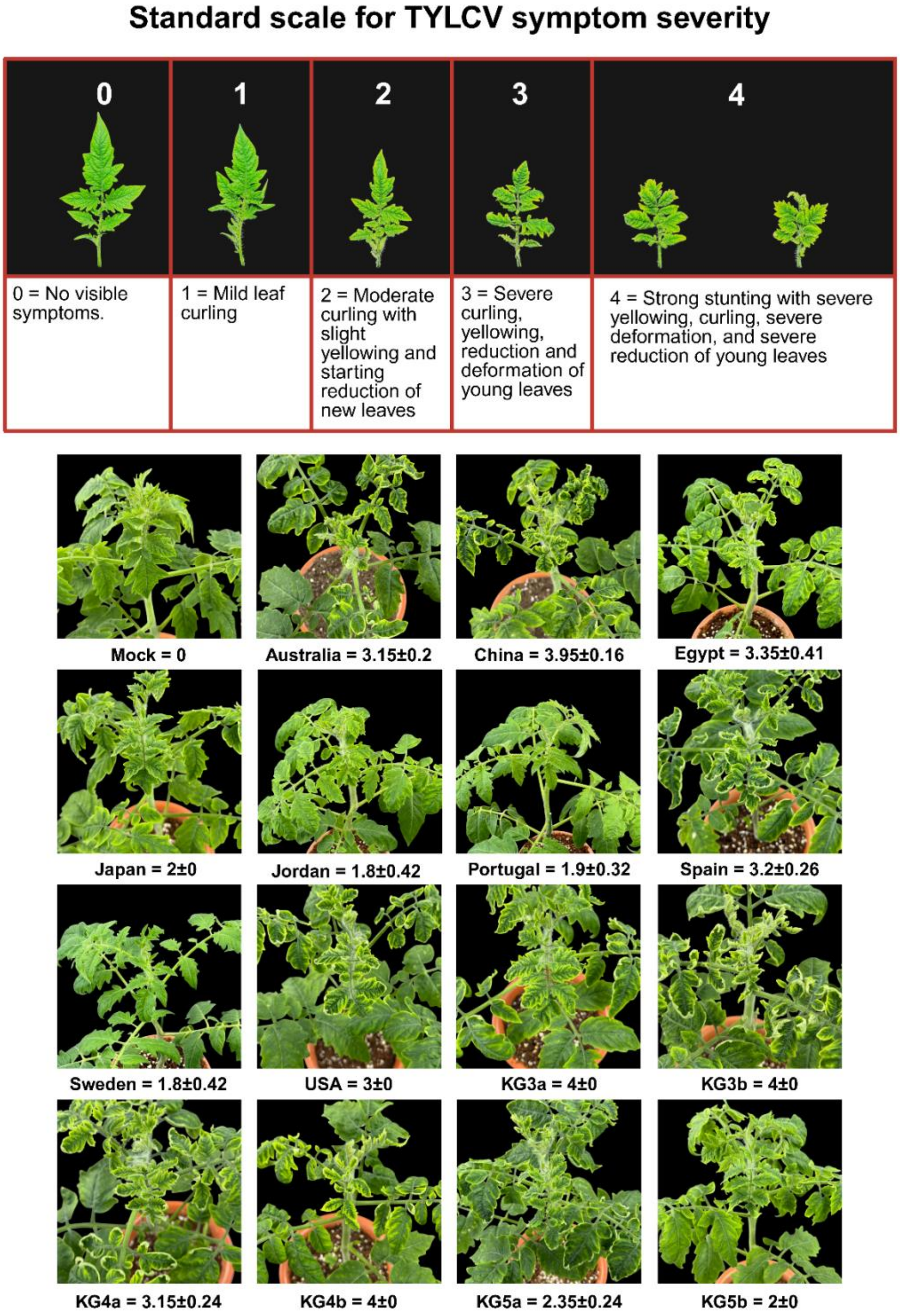

Supplemental Figure 7. Representative symptom severity scale (0–4) of TYLCV-infected tomato plants. TYLCV-infected MM plants in this study corresponding to each score are shown below the schematic scale. Images were captured at 21 days post-inoculation (dpi) under controlled greenhouse conditions.

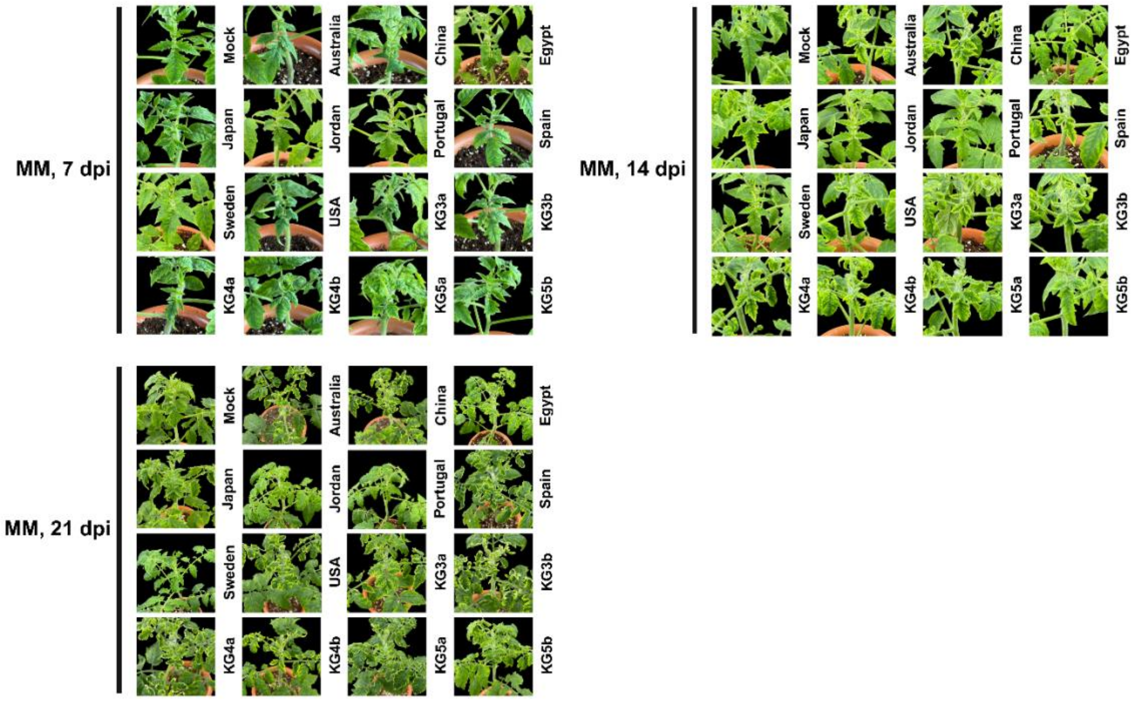

Supplemental Figure 8. Symptom development and validation of DeepTYLCV-predicted severity classes in MM plants. At 7 days post-inoculation (dpi), early infection symptoms included upward leaf curling and mild yellowing in isolates predicted as severe, while mild isolates showed slight or no visible symptoms. At 14 dpi, severe isolates exhibited pronounced curling, apical reduction, and yellowing, whereas mild isolates maintained moderate symptoms. By 21 dpi, severe isolates showed stunted growth, strong leaf curling, and deformation.

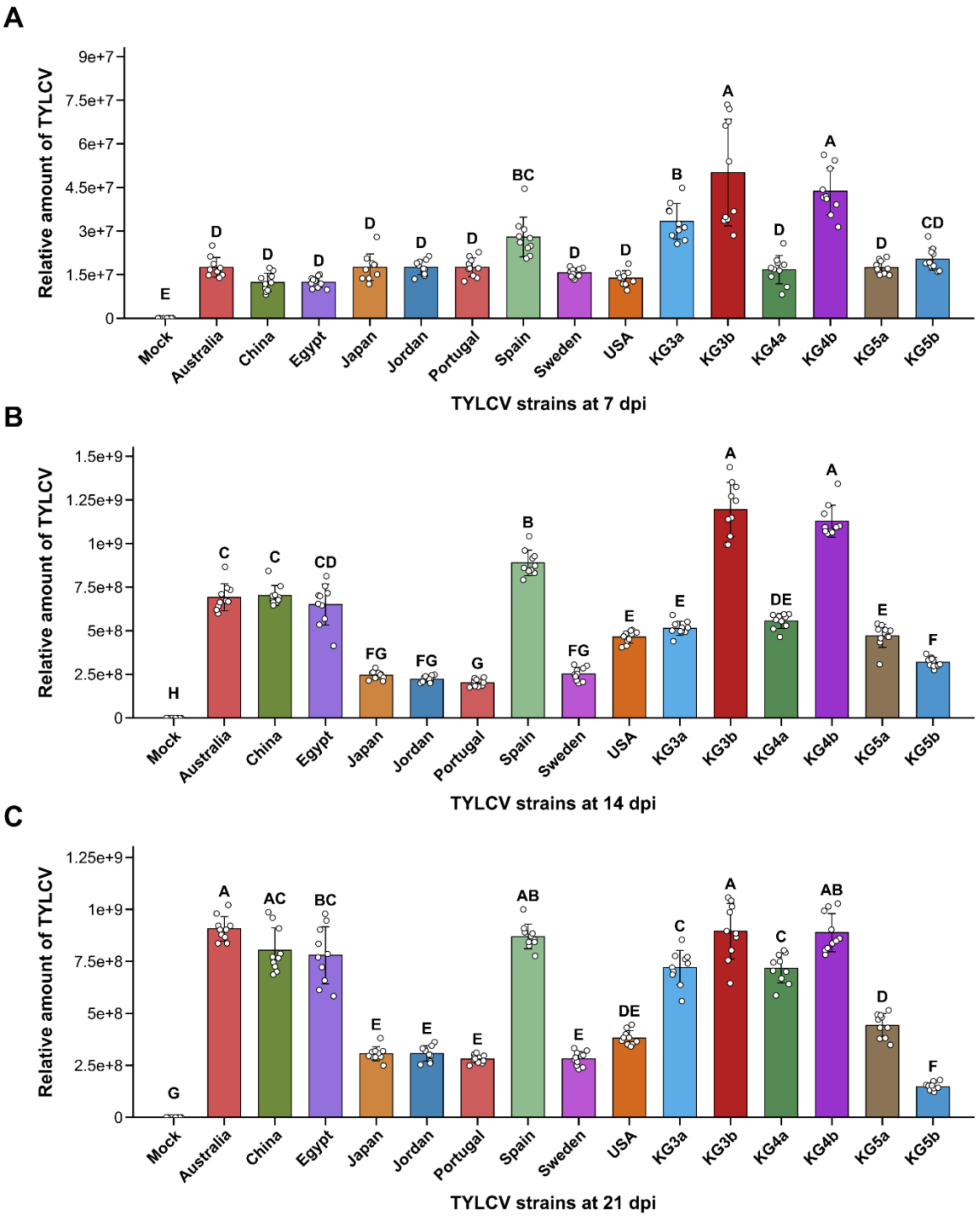

Supplemental Figure 9. Quantitative analysis of viral accumulation in tomato plants inoculated with 15 TYLCV isolates. Distinct letters above bars indicate statistically significant differences between isolates according to Tukey's multiple comparisons test ($p < 0.05$) according to one-way ANOVA analysis. (A) At 7 dpi, One-way ANOVA

showed a significant overall effect of isolate on viral accumulation ($F_{15,\,144} = 41.167, p < 0.001, \eta^2 = 0.8109$). (B) At 14 dpi, One-way ANOVA showed a significant overall effect of isolate on viral accumulation ($F_{15,\,144} = 244.714, p < 0.001, \eta^2 = 0.9623$). (C) At 21 dpi, One-way ANOVA showed a significant overall effect of isolate on viral accumulation ($F_{15,\,144} = 176.963, p < 0.001, \eta^2 = 0.9485$). All TYLCV-inoculated plants showed significantly higher viral accumulation than the mock control ($p < 0.001$). Severe isolates (e.g., TYLCV-Australia, -China, -Egypt, -Spain, -USA, -KG3a, -KG3b, -KG4a, and -KG4b) accumulated higher viral loads than mild isolates (TYLCV-Japan, -Jordan, -Portugal, -Sweden, -KG5a, and -KG5b) across all timepoints.

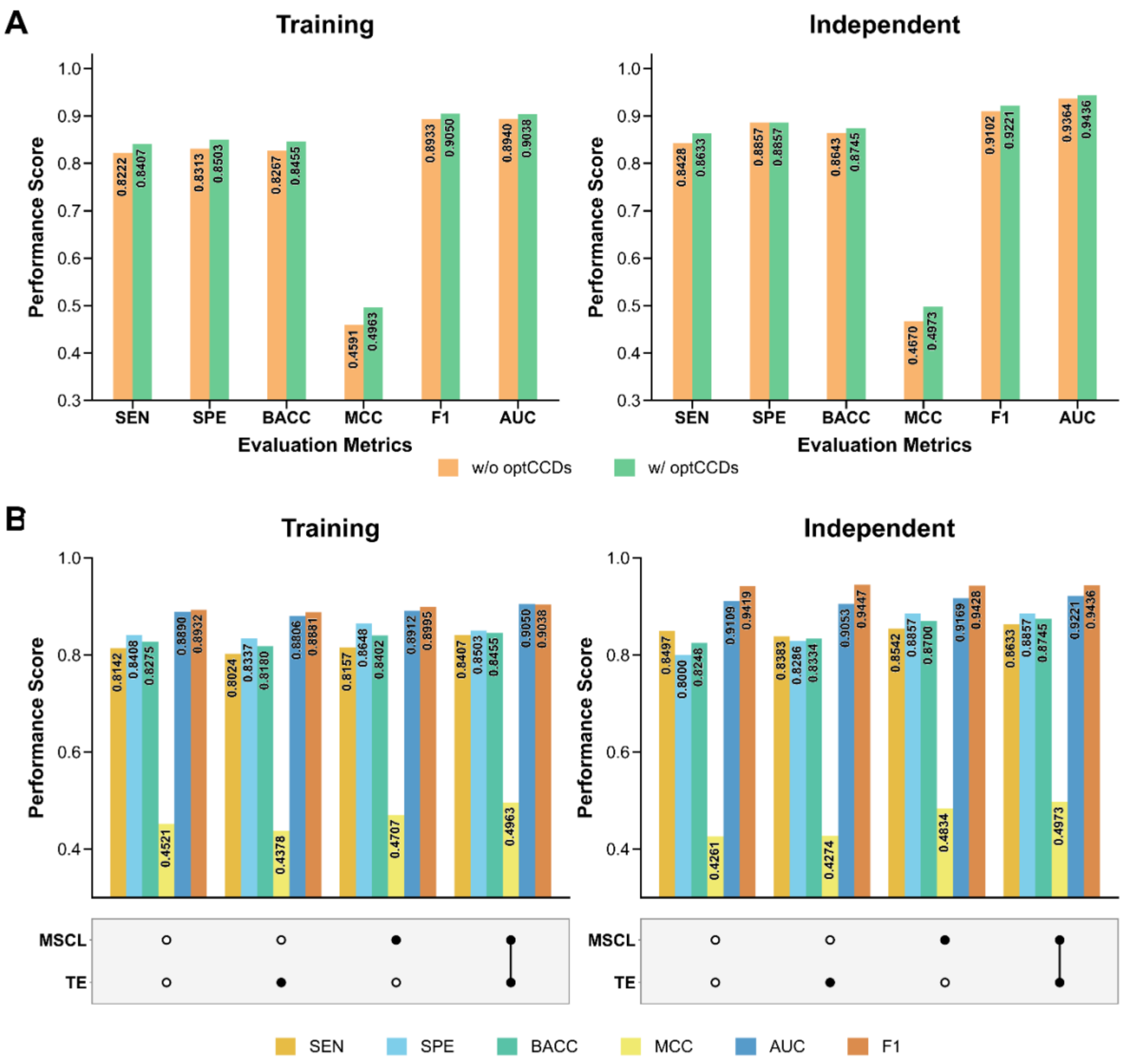

Supplemental Figure 10. Ablation analysis of DeepTYLCV model components. (A) This ablation experiment assesses how integrating Top-3 PLM-based feature representations with optCCDs affects model performance on training and independent datasets. The orange bar shows performance based only on Top-3 PLM-based embeddings, while the light green bar shows performance with the combined Top-3 embeddings and optCCDs. (B) The other ablation experiment examines the contribution of the TE and MSCL. The presence or absence of each module is shown in the Upset-style matrix below each bar plot conducted on the training and independent test sets.

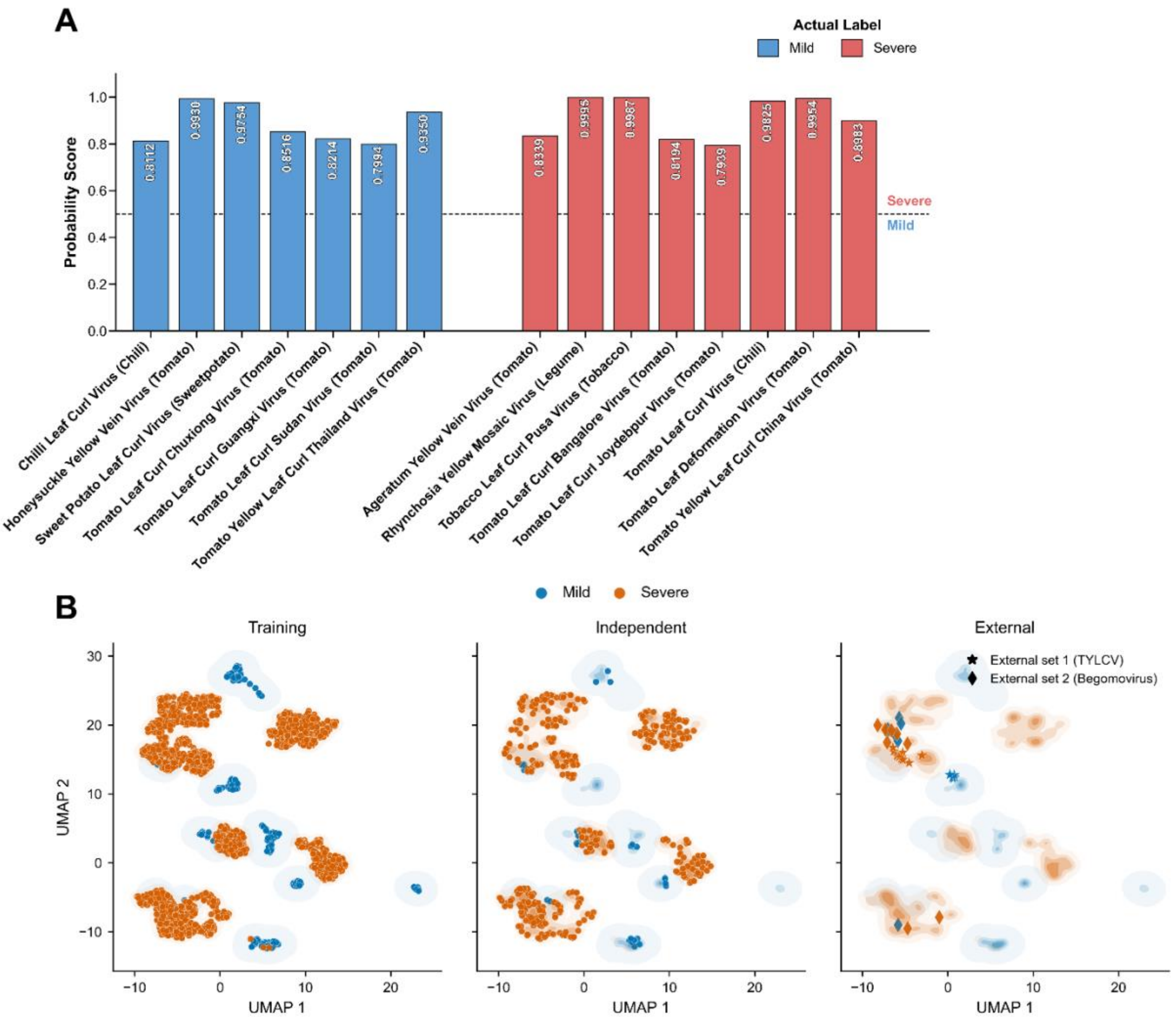

Supplemental Figure 11. Transferability evaluation on external TYLCV and begomoviruses by DeepTYLCV. (A) The bar chart shows the predicted severity scores for each genome. The horizontal dashed line at 0.5 indicates the classification threshold. Bar colors indicate the actual known phenotypes of the isolates ($blue = mild$, $red = severe$). DeepTYLCV predicted all tested genomes as severe ($score >= 0.5$), thereby misclassifying the actual mild isolates. Virus names and their hosts are provided on the x-axis. (B) UMAP visualization of DeepTYLCV's latent feature representations across training, independent, and external validation datasets. In the left panel, the UMAP projection of the training set shows that severe and mild isolates form distinct, well-separated clusters, indicating that DeepTYLCV effectively extracts

discriminative features for phenotypic classification. The middle panel displays the feature distribution of the independent test set, showing that the representations maintain the clear spatial separation between severe and mild phenotypes established during training. The right panel presents the UMAP projection of External Set 1 (TYLCV isolates) and External Set 2 (non-TYLCV begomovirus species). While External Set 1's distribution aligns with the established severe and mild clusters, all samples from External Set 2, regardless of actual phenotype, map to the severe latent space defined during training, resulting in systematic overprediction of severity for broader begomoviruses.

Supplemental Table 4. Summary of collected TYLCV isolates for model training.

No	Accession number	Country	Year (Collected)	Host	Size (bp)	Note	Severity
1	KX347140.1	Australia	2010	Tomato	2781		Severe
2	KX347139.1	Australia	2010	Tomato	2781		Severe
3	KX347145.1	Australia	2010	Tomato	2781		Severe
4	KX347143.1	Australia	2010	Tomato	2781		Severe
5	KX347144.1	Australia	2010	Tomato	2781		Severe
6	KX347142.1	Australia	2010	Tomato	2781		Severe
7	KX347141.1	Australia	2010	Tomato	2782		Severe
8	KX347138.1	Australia	2009	Tomato	2781		Severe
9	KX347133.1	Australia	2009	Tomato	2781		Severe
10	KX347132.1	Australia	2009	Tomato	2781		Severe
11	KX347136.1	Australia	2009	Tomato	2781		Severe
12	KX347135.1	Australia	2009	Tomato	2781		Severe
13	KX347137.1	Australia	2009	Tomato	2781		Severe
14	KX347134.1	Australia	2009	Tomato	2781		Severe
15	KX347131.1	Australia	2009	Tomato	2781		Severe
16	KX347129.1	Australia	2007	Tomato	2781		Severe
17	KX347130.1	Australia	2007	Tomato	2781		Severe
18	KX347128.1	Australia	2007	Tomato	2781		Severe
19	KX347121.1	Australia	2006	Tomato	2781		Severe
20	KX347118.1	Australia	2006	Tomato	2781		Severe
21	KX347097.1	Australia	2006	Tomato	2781		Severe
22	KX347096.1	Australia	2006	Tomato	2781		Severe
23	KX347105.1	Australia	2006	Tomato	2781		Severe
24	KX347110.1	Australia	2006	Tomato	2781		Severe
25	KX347108.1	Australia	2006	Tomato	2781		Severe
26	KX347111.1	Australia	2006	Tomato	2781		Severe
27	KX347106.1	Australia	2006	Tomato	2781		Severe
28	KX347098.1	Australia	2006	Tomato	2781		Severe
29	KX347103.1	Australia	2006	Tomato	2781		Severe
30	KX347117.1	Australia	2006	Tomato	2781		Severe
31	KX347107.1	Australia	2006	Tomato	2781		Severe
32	KX347115.1	Australia	2006	Tomato	2781		Severe
33	KX347112.1	Australia	2006	Tomato	2781		Severe

34	KX347120.1	Australia	2006	Tomato	2781		Severe
35	KX347113.1	Australia	2006	Tomato	2781		Severe
36	KX347114.1	Australia	2006	Tomato	2781		Severe
37	KX347104.1	Australia	2006	Tomato	2781		Severe
38	KX347116.1	Australia	2006	Tomato	2781		Severe
39	KX347101.1	Australia	2006	Tomato	2781		Severe
40	GU178816.1	Australia	2006	Tomato	2781		Severe
41	KX347102.1	Australia	2006	Tomato	2781		Severe
42	KX347100.1	Australia	2006	Tomato	2781		Severe
43	KX347099.1	Australia	2006	Tomato	2781		Severe
44	GU178814.1	Australia	2006	Tomato	2769		Severe
45	GU178813.1	Australia	2006	Tomato	2769		Severe
46	KX347119.1	Australia	2006	Tomato	2781		Severe
47	KX347109.1	Australia	2006	Tomato	2781		Severe
48	KX347095.1	Australia	2006	Tomato	2781		Severe
49	GU178818.1	Australia	2006	Tomato	2781		Severe
50	GU178815.1	Australia	2006	Tomato	2780		Severe
51	KX347127.1	Australia	2006	Tomato	2781		Severe
52	GU178819.1	Australia	2006	Tomato	2781		Severe
53	KX347125.1	Australia	2006	Tomato	2781		Severe
54	KX347124.1	Australia	2006	Tomato	2781		Severe
55	GU178820.1	Australia	2006	Tomato	2781		Severe
56	KX347126.1	Australia	2006	Tomato	2781		Severe
57	GU178817.1	Australia	2006	Tomato	2781		Severe
58	KX347123.1	Australia	2006	Tomato	2781		Severe
59	KX347122.1	Australia	2006	Tomato	2781		Severe
60	KX347094.1	Australia	2003	Tomato	2781		Severe
61	KY412552.1	Azerbaijan	2014	Tomato	2783		Severe
62	PP438362.1	China	2023	Tomato	2781		Severe
63	PP438356.1	China	2023	Tomato	2781		Severe
64	PP438341.1	China	2023	Tomato	2780		Severe
65	PP438342.1	China	2023	Tomato	2781		Severe
66	PP438359.1	China	2023	Tomato	2781		Severe
67	PP438358.1	China	2023	Tomato	2781		Severe
68	PP438353.1	China	2023	Tomato	2781		Severe
69	PP438354.1	China	2023	Tomato	2771		Severe

70	PP438363.1	China	2023	Tomato	2771		Severe
71	PP438351.1	China	2023	Tomato	2768		Severe
72	PP438352.1	China	2023	Tomato	2768		Severe
73	PP438355.1	China	2023	Tomato	2768		Severe
74	PP438361.1	China	2023	Tomato	2768		Severe
75	PP438357.1	China	2023	Tomato	2757		Severe
76	PP438343.1	China	2022	Tomato	2781		Severe
77	PP438344.1	China	2022	Tomato	2781		Severe
78	PP438348.1	China	2022	Tomato	2781		Severe
79	PP438345.1	China	2022	Tomato	2781		Severe
80	PP438347.1	China	2022	Tomato	2781		Severe
81	PP438346.1	China	2022	Tomato	2781		Severe
82	OQ428182.1	China	2022	Tobacco	2781		Severe
83	PP274946.1	China	2022	Tomato	2781		Severe
84	PP438360.1	China	2022	Tomato	2780		Severe
85	PP438340.1	China	2022	Tomato	2768		Severe
86	PP438349.1	China	2022	Tomato	2771		Severe
87	OR670949.1	China	2022	Tomato	2771		Severe
88	PP438350.1	China	2022	Tomato	2757		Severe
89	PP438339.1	China	2021	Tomato	2781		Severe
90	ON093143.1	China	2021	Tomato	2781		Severe
91	ON093139.1	China	2021	Tomato	2781		Severe
92	PP259080.1	China	2021	Tomato	2781		Severe
93	ON093144.1	China	2021	Tomato	2781		Severe
94	ON093152.1	China	2021	Tomato	2781		Severe
95	ON093142.1	China	2021	Tomato	2768		Severe
96	ON093146.1	China	2021	Tomato	2768		Severe
97	ON093145.1	China	2021	Tomato	2768		Severe
98	ON093148.1	China	2021	Tomato	2768		Severe
99	ON093149.1	China	2021	Tomato	2768		Severe
100	ON093150.1	China	2021	Tomato	2768		Severe
101	ON093153.1	China	2021	Tomato	2768		Severe
102	ON093151.1	China	2021	Tomato	2768		Severe
103	ON093140.1	China	2021	Tomato	2781		Severe
104	ON093141.1	China	2021	Tomato	2781		Severe
105	PP259079.1	China	2021	Tomato	2781		Severe

106	ON093147.1	China	2021	Tomato	2781		Severe
107	PP259078.1	China	2021	Tomato	2757		Severe
108	MW779532.1	China	2020	Tomato	2781		Severe
109	MW779534.1	China	2020	Tomato	2781		Severe
110	MW678600.1	China	2020	Tomato	2781		Severe
111	MW779533.1	China	2020	Tomato	2781		Severe
112	MW779530.1	China	2020	Tomato	2781		Severe
113	MW779531.1	China	2020	Tomato	2781		Severe
114	MW678599.1	China	2020	Tomato	2752		Severe
115	MN842307.1	China	2019	Tomato	2781		Severe
116	MN842308.1	China	2019	Tomato	2781		Severe
117	MN842311.1	China	2019	Tomato	2781		Severe
118	MN842312.1	China	2019	Tomato	2781		Severe
119	MK908814.1	China	2019	Tomato	2781		Severe
120	MK908813.1	China	2019	Tomato	2781		Severe
121	MW735448.1	China	2019	Tomato	2781		Severe
122	MW735449.1	China	2019	Tomato	2781		Severe
123	MT375611.1	China	2019	Tomato	2781		Severe
124	MT375612.1	China	2019	Tomato	2781		Severe
125	MT375613.1	China	2019	Tomato	2781		Severe
126	MT375609.1	China	2019	Tomato	2781		Severe
127	MN432609.1	China	2019	Tomato	2781		Severe
128	MK908815.1	China	2019	Tomato	2781		Severe
129	MW735451.1	China	2019	Tomato	2781		Severe
130	MW779529.1	China	2019	Tomato	2781		Severe
131	OR544985.1	China	2019	Zucchini	2781		Severe
132	OR544986.1	China	2019	Zucchini	2781		Severe
133	MW735450.1	China	2019	Tomato	2780		Severe
134	MW286511.1	China	2019	Tomato	2781		Severe
135	MN842313.1	China	2019	Tomato	2781		Severe
136	MN842314.1	China	2019	Tomato	2781		Severe
137	MN842309.1	China	2019	Tomato	2769		Severe
138	MN842310.1	China	2019	Tomato	2769		Severe
139	MW735447.1	China	2019	Tomato	2781		Severe
140	MW735446.1	China	2019	Tomato	2781		Severe
141	MW389933.1	China	2019	Tomato	2782		Severe

142	MT375608.1	China	2019	Tomato	2776		Severe
143	MW779528.1	China	2019	Tomato	2781		Severe
144	MT863567.1	China	2019	Tomato	2781		Severe
145	MT969010.1	China	2019	Tomato	2757		Severe
146	MW389934.1	China	2019	Tomato	2752		Severe
147	MT375610.1	China	2019	Tomato	2752		Severe
148	MN381713.1	China	2018	Pumpkin	2781		Severe
149	MN910280.1	China	2018	Tomato	2781		Severe
150	MN520591.1	China	2018	Tomato	2778		Severe
151	MN966542.1	China	2018	Tomato	2778		Severe
152	MN966544.1	China	2018	Tomato	2781		Severe
153	MN966545.1	China	2018	Tomato	2781		Severe
154	MN966546.1	China	2018	Tomato	2781		Severe
155	MN503143.1	China	2018	Tomato	2781		Severe
156	MN503144.1	China	2018	Tomato	2781		Severe
157	MN966547.1	China	2018	Tomato	2781		Severe
158	MN966552.1	China	2018	Tomato	2781		Severe
159	MN503133.1	China	2018	Tomato	2781		Severe
160	MN503141.1	China	2018	Tomato	2781		Severe
161	MN520579.1	China	2018	Tomato	2781		Severe
162	MN520578.1	China	2018	Tomato	2781		Severe
163	MN503155.1	China	2018	Tomato	2781		Severe
164	MN520589.1	China	2018	Tomato	2781		Severe
165	MN966555.1	China	2018	Tomato	2781		Severe
166	MN503132.1	China	2018	Tomato	2781		Severe
167	MN503138.1	China	2018	Tomato	2781		Severe
168	MN520590.1	China	2018	Tomato	2781		Severe
169	MN503134.1	China	2018	Tomato	2781		Severe
170	MN520584.1	China	2018	Tomato	2781		Severe
171	MN503146.1	China	2018	Tomato	2781		Severe
172	MN520581.1	China	2018	Tomato	2781		Severe
173	MN520580.1	China	2018	Tomato	2781		Severe
174	MN503151.1	China	2018	Tomato	2781		Severe
175	MN520587.1	China	2018	Tomato	2781		Severe
176	MN966553.1	China	2018	Tomato	2781		Severe
177	MN520586.1	China	2018	Tomato	2781		Severe

178	MN520585.1	China	2018	Tomato	2781		Severe
179	MN503136.1	China	2018	Tomato	2781		Severe
180	MN503147.1	China	2018	Tomato	2781		Severe
181	MN503148.1	China	2018	Tomato	2781		Severe
182	MN503142.1	China	2018	Tomato	2781		Severe
183	MN520583.1	China	2018	Tomato	2781		Severe
184	MN966554.1	China	2018	Tomato	2781		Severe
185	MN966549.1	China	2018	Tomato	2781		Severe
186	MN503131.1	China	2018	Tomato	2781		Severe
187	MN503137.1	China	2018	Tomato	2781		Severe
188	MN503154.1	China	2018	Tomato	2781		Severe
189	MN503152.1	China	2018	Tomato	2781		Severe
190	MN520588.1	China	2018	Tomato	2781		Severe
191	MN503140.1	China	2018	Tomato	2781		Severe
192	MN503135.1	China	2018	Tomato	2781		Severe
193	MN520577.1	China	2018	Tomato	2782		Severe
194	MN520582.1	China	2018	Tomato	2781		Severe
195	MN520575.1	China	2018	Tomato	2781		Severe
196	MN520576.1	China	2018	Tomato	2781		Severe
197	MN503145.1	China	2018	Tomato	2781		Severe
198	MN503139.1	China	2018	Tomato	2781		Severe
199	MN503149.1	China	2018	Tomato	2780		Severe
200	MN966551.1	China	2018	Tomato	2781		Severe
201	MN503150.1	China	2018	Tomato	2781		Severe
202	MN520593.1	China	2018	Tomato	2779		Severe
203	MN520592.1	China	2018	Tomato	2779		Severe
204	MN520596.1	China	2018	Tomato	2779		Severe
205	MN520595.1	China	2018	Tomato	2779		Severe
206	MN520594.1	China	2018	Tomato	2779		Severe
207	MN503153.1	China	2018	Tomato	2764		Severe
208	OP186933.1	China	2018	Tomato	2781		Severe
209	MT084806.1	China	2018	Apple	2730		Severe
210	MN966548.1	China	2018	Tomato	2776		Severe
211	MN966550.1	China	2018	Tomato	2776		Severe
212	MK562703.1	China	2017	Tomato	2781		Severe
213	MH703582.1	China	2017	Tomato	2781		Severe

214	MK495362.1	China	2017	Weed	2780		Severe
215	MN966540.1	China	2017	Weed	2778		Severe
216	MN966541.1	China	2017	Weed	2778		Severe
217	MN966543.1	China	2017	Weed	2778		Severe
218	MN503090.1	China	2017	Tomato	2778		Severe
219	MN503095.1	China	2017	Tomato	2778		Severe
220	MN503096.1	China	2017	Tomato	2778		Severe
221	MN503089.1	China	2017	Tomato	2778		Severe
222	MN503091.1	China	2017	Tomato	2781		Severe
223	MN503099.1	China	2017	Tomato	2781		Severe
224	MN503092.1	China	2017	Tomato	2781		Severe
225	MN503093.1	China	2017	Tomato	2781		Severe
226	MN503094.1	China	2017	Tomato	2781		Severe
227	MN503097.1	China	2017	Tomato	2781		Severe
228	MN503102.1	China	2017	Tomato	2781		Severe
229	MN503103.1	China	2017	Tomato	2781		Severe
230	MN503118.1	China	2017	Tomato	2781		Severe
231	MN503100.1	China	2017	Tomato	2781		Severe
232	MN503113.1	China	2017	Tomato	2781		Severe
233	MN503126.1	China	2017	Tomato	2781		Severe
234	MN503125.1	China	2017	Tomato	2781		Severe
235	MN503114.1	China	2017	Tomato	2781		Severe
236	MN503115.1	China	2017	Tomato	2781		Severe
237	MN503107.1	China	2017	Tomato	2781		Severe
238	MN503120.1	China	2017	Tomato	2781		Severe
239	MN503111.1	China	2017	Tomato	2781		Severe
240	MN503112.1	China	2017	Tomato	2781		Severe
241	MN503098.1	China	2017	Tomato	2781		Severe
242	MN503117.1	China	2017	Tomato	2781		Severe
243	MN503119.1	China	2017	Tomato	2781		Severe
244	MN503121.1	China	2017	Tomato	2781		Severe
245	MN503122.1	China	2017	Tomato	2781		Severe
246	MN503123.1	China	2017	Tomato	2781		Severe
247	MN503124.1	China	2017	Tomato	2781		Severe
248	MN503128.1	China	2017	Tomato	2781		Severe
249	MN503106.1	China	2017	Tomato	2781		Severe

250	MN503104.1	China	2017	Tomato	2781		Severe
251	MN503105.1	China	2017	Tomato	2781		Severe
252	MN503101.1	China	2017	Tomato	2781		Severe
253	MN503116.1	China	2017	Tomato	2781		Severe
254	MN503109.1	China	2017	Tomato	2781		Severe
255	MN503110.1	China	2017	Tomato	2781		Severe
256	MN503108.1	China	2017	Tomato	2781		Severe
257	MK495363.1	China	2017	Weed	2781		Severe
258	MN503127.1	China	2017	Tomato	2781		Severe
259	MN503129.1	China	2017	Tomato	2781		Severe
260	MN503130.1	China	2017	Tomato	2779		Severe
261	MF668139.1	China	2017	Tomato	2781		Severe
262	MF668140.1	China	2017	Tomato	2780		Severe
263	MF668141.1	China	2017	Tomato	2781		Severe
264	MK558201.1	China	2016	Tomato	2781		Severe
265	MH205950.1	China	2016	Bean	2781		Severe
266	MK558200.1	China	2016	Tomato	2781		Severe
267	MF590741.1	China	2016	Tomato	2781		Severe
268	MF590742.1	China	2016	Tomato	2781		Severe
269	MF590737.1	China	2016	Tomato	2781		Severe
270	MF590735.1	China	2016	Tomato	2781		Severe
271	MF590739.1	China	2016	Tomato	2781		Severe
272	MF590734.1	China	2016	Tomato	2781		Severe
273	MF590738.1	China	2016	Tomato	2781		Severe
274	MF590740.1	China	2016	Tomato	2781		Severe
275	KX034552.1	China	2016	Tomato	2781		Severe
276	MF590733.1	China	2016	Tomato	2776		Severe
277	MF590732.1	China	2016	Tomato	2781		Severe
278	MF590736.1	China	2016	Tomato	2780		Severe
279	MK562702.1	China	2015	Tomato	2781		Severe
280	KT338295.1	China	2015	Tomato	2781		Severe
281	KU760892.1	China	2015	Pepper	2781		Severe
282	KT338296.1	China	2015	Tomato	2768		Severe
283	KU760889.1	China	2015	Pepper	2781		Severe
284	KX034553.1	China	2015	Tomato	2781		Severe
285	KU760888.1	China	2015	Pepper	2781		Severe

286	KU760890.1	China	2015	Pepper	2781		Severe
287	KU760891.1	China	2015	Pepper	2781		Severe
288	MN503087.1	China	2014	Tomato	2777		Severe
289	MN503088.1	China	2014	Tomato	2781		Severe
290	KP684146.1	China	2014	Weed	2781		Severe
291	KU975399.1	China	2014	Tomato	2781		Severe
292	KT338294.1	China	2014	Tomato	2740		Severe
293	KU934104.1	China	2014	Tomato	2781		Severe
294	KU975398.1	China	2014	Tomato	2781		Severe
295	KU975397.1	China	2014	Tomato	2781		Severe
296	KU975396.1	China	2014	Tomato	2775		Severe
297	KT338293.1	China	2014	Tomato	2781		Severe
298	KT852577.1	China	2014	Tomato	2781		Severe
299	KJ850344.1	China	2014	Tomato	2781		Severe
300	KP685598.1	China	2014	Tomato	2781		Severe
301	KM435326.1	China	2014	Tomato	2781		Severe
302	KM435328.1	China	2014	Tomato	2781		Severe
303	KM435324.1	China	2014	Tomato	2781		Severe
304	KM435322.1	China	2014	Tomato	2781		Severe
305	KM435320.1	China	2014	Tomato	2781		Severe
306	KU892717.1	China	2013	Tomato	2781		Severe
307	KF906542.1	China	2013	Tomato	2781		Severe
308	KJ754188.1	China	2013	Tomato	2781		Severe
309	KJ754193.1	China	2013	Perilla	2781		Severe
310	KJ754189.1	China	2013	Tomato	2781		Severe
311	KJ754186.1	China	2013	Tomato	2781		Severe
312	KJ754187.1	China	2013	Tomato	2781		Severe
313	KJ546418.1	China	2013	Tomato	2781		Severe
314	KC999850.1	China	2013	Pepper	2781		Severe
315	KJ754191.1	China	2013	Tomato	2781		Severe
316	KC999849.1	China	2013	Pepper	2781		Severe
317	KJ754190.1	China	2013	Tomato	2781		Severe
318	KM506950.1	China	2013	Tomato	2781		Severe
319	KJ754194.1	China	2013	Tomato	2781		Severe
320	KM506951.1	China	2013	Tomato	2781		Severe
321	KF990604.1	China	2013	Bitter bine	2781		Severe

322	KJ754192.1	China	2013	Tomato	2781		Severe
323	KM506948.1	China	2013	Tomato	2781		Severe
324	KM506947.1	China	2013	Tomato	2781		Severe
325	KM506953.1	China	2013	Tomato	2781		Severe
326	KM506958.1	China	2013	Tomato	2781		Severe
327	KM506957.1	China	2013	Tomato	2781		Severe
328	KM506955.1	China	2013	Tomato	2781		Severe
329	KM506949.1	China	2013	Tomato	2781		Severe
330	KF356163.1	China	2013	Tomato	2781		Severe
331	KM506956.1	China	2013	Tomato	2780		Severe
332	KM506954.1	China	2013	Tomato	2780		Severe
333	KM506952.1	China	2013	Tomato	2781		Severe
334	KJ125411.1	China	2013	Tomato	2779		Severe
335	KM435325.1	China	2013	Tomato	2781		Severe
336	KM435327.1	China	2013	Tomato	2781		Severe
337	KM435323.1	China	2013	Tomato	2781		Severe
338	KM435321.1	China	2013	Tomato	2781		Severe
339	KM435319.1	China	2013	Tomato	2781		Severe
340	KJ125410.1	China	2012	Tomato	2781		Severe
341	KF612971.1	China	2012	Tomato	2781		Severe
342	KC702796.1	China	2012	Tomato	2781		Severe
343	KC999847.1	China	2012	Tomato	2781		Severe
344	KC999846.1	China	2012	Tomato	2781		Severe
345	KC999848.1	China	2012	Tomato	2781		Severe
346	KC852151.1	China	2012	Zinnia	2781		Severe
347	KC852147.1	China	2012	Cotton	2781		Severe
348	KC999844.1	China	2012	Tomato	2781		Severe
349	KC852150.1	China	2012	Weed	2781		Severe
350	JQ807735.1	China	2012	Tomato	2781		Severe
351	KC999843.1	China	2012	Pepper	2781		Severe
352	KC852149.1	China	2012	Weed	2781		Severe
353	KC999845.1	China	2012	Tomato	2781		Severe
354	KC810892.1	China	2012	Tomato	2781		Severe
355	JX128099.1	China	2012	Tomato	2781		Severe
356	KM506961.1	China	2012	Tomato	2781		Severe
357	JX997803.1	China	2012	Hollyhock	2781		Severe

358	JX997801.1	China	2012	Cowpea	2781		Severe
359	JX997802.1	China	2012	Hollyhock	2781		Severe
360	JX997799.1	China	2012	Tomato	2781		Severe
361	JX997800.1	China	2012	Cowpea	2781		Severe
362	KC702797.1	China	2012	Tomato	2781		Severe
363	JQ004048.1	China	2011	Tomato	2781		Severe
364	JQ038238.1	China	2011	Tomato	2781		Severe
365	KC999851.1	China	2011	Amaranth	2781		Severe
366	JQ004052.1	China	2011	Tomato	2781		Severe
367	JQ038237.1	China	2011	Tomato	2781		Severe
368	JQ004049.1	China	2011	Tomato	2781		Severe
369	JQ004045.1	China	2011	Tomato	2781		Severe
370	JQ038234.1	China	2011	Tomato	2781		Severe
371	JN990922.1	China	2011	Tomato	2781		Severe
372	JQ038239.1	China	2011	Tomato	2781		Severe
373	JQ038235.1	China	2011	Tomato	2781		Severe
374	JQ038236.1	China	2011	Tomato	2781		Severe
375	JN990924.1	China	2011	Tomato	2781		Severe
376	JQ004051.1	China	2011	Tomato	2780		Severe
377	JX070043.1	China	2011	Tomato	2781		Severe
378	JQ411237.1	China	2011	Pepper	2781		Severe
379	JQ038240.1	China	2011	Tomato	2781		Severe
380	JX456638.1	China	2011	Tomato	2781		Severe
381	JX456639.1	China	2011	Tomato	2781		Severe
382	JX070044.1	China	2011	Tomato	2781		Severe
383	JQ867092.1	China	2011	Tomato	2781		Severe
384	JX456644.1	China	2011	Datura	2781		Severe
385	JQ038233.1	China	2011	Tomato	2781		Severe
386	JX456640.1	China	2011	Tomato	2781		Severe
387	JX456643.1	China	2011	Eggplant	2781		Severe
388	JX456637.1	China	2011	Tomato	2781		Severe
389	JX456641.1	China	2011	Pepper	2781		Severe
390	JX456642.1	China	2011	Pepper	2781		Severe
391	JQ004046.1	China	2011	Tomato	2781		Severe
392	JN990923.1	China	2011	Tomato	2781		Severe
393	JX997798.1	China	2011	Tomato	2781		Severe

394	JX070042.1	China	2011	Tomato	2781		Severe
395	JN990927.1	China	2011	Tomato	2781		Severe
396	JX070045.1	China	2011	Tomato	2781		Severe
397	KC138546.1	China	2011	Tomato	2781		Severe
398	KC138545.1	China	2011	Tomato	2781		Severe
399	JN990928.1	China	2011	Tomato	2781		Severe
400	KM506960.1	China	2011	Tomato	2781		Severe
401	KM506959.1	China	2011	Tomato	2781		Severe
402	JN990925.1	China	2011	Tomato	2781		Severe
403	JQ004050.1	China	2011	Tomato	2781		Severe
404	JQ326957.1	China	2011	Common bean	2781		Severe
405	KC138543.1	China	2011	Tomato	2781		Severe
406	KC138544.1	China	2011	Tomato	2781		Severe
407	JQ004047.1	China	2011	Tomato	2781		Severe
408	JN990926.1	China	2011	Tomato	2781		Severe
409	JQ038232.1	China	2011	Tomato	2781		Severe
410	JX128100.1	China	2011	Tomato	2781		Severe
411	JN412854.1	China	2011	Tomato	2781		Severe
412	HM627884.1	China	2010	Tomato	2781		Severe
413	HM627885.1	China	2010	Tomato	2781		Severe
414	FN650807.1	China	2010	Tomato	2781		Severe
415	FN650808.1	China	2010	Tomato	2781		Severe
416	HM627881.1	China	2010	Tomato	2781		Severe
417	HM627880.1	China	2009	Tomato	2781		Severe
418	HM208334.1	China	2009	Whitefly	2781		Severe
419	HM627883.1	China	2009	Tomato	2781		Severe
420	HM627882.1	China	2009	Tomato	2781		Severe
421	GU434142.1	China	2008	Tomato	2781		Severe
422	AM698117.1	China	2008	Tomato	2781		Severe
423	GU199587.1	China	2008	Tomato	2781		Severe
424	FN256257.1	China	2008	Tomato	2781		Severe
425	GU434141.1	China	2008	Tomato	2781		Severe
426	AM698118.1	China	2008	Tomato	2781		Severe
427	FN256259.1	China	2008	Tomato	2781		Severe
428	AM698119.1	China	2008	Tomato	2781		Severe
429	GU111505.1	China	2007	Tomato	2781		Severe

430	FN256258.1	China	2007	Tomato	2781		Severe
431	FN252890.1	China	2007	Tomato	2781		Severe
432	FN256256.1	China	2007	Tomato	2781		Severe
433	EU031444.1	China	2007	-	2781		Severe
434	AM282874.1	China	2006	Tomato	2781		Severe
435	JX910534.1	China	2012	Asian copperleaf	2781 bp		Mild
436	MW807604.1	Costa Rica	2015	Tomato	2781		Severe
437	MW654012.1	Costa Rica	2015	Tomato	2781		Severe
438	KY064016.1	Costa Rica	2012	Tomato	2781		Severe
439	KF533857.1	Costa Rica	2012	Tomato	2781		Severe
440	KF533855.1	Costa Rica	2012	Tomato	2781		Severe
441	KF533856.1	Costa Rica	2012	Tomato	2781		Severe
442	KM926626.1	Cuba	2011	Tomato	2781		Severe
443	KM926625.1	Cuba	2011	Tomato	2781		Severe
444	AJ223505.1	Cuba	2005	-	2781		Severe
445	AF024715.2	Dominican Republic	2003	-	2781		Severe
446	KJ913683.1	Dominican Republic	2011	Toamto	2791 bp		Mild
447	KJ913682.1	Dominican Republic	2011	Toamto	2791 bp		Mild
448	AY594174.1	Egypt	2006	-	2781		Severe
449	EF107520.1	Egypt	2000	-	2784		Severe
450	HF548826.1	Estonia	2008	Tomato	2787		Severe
451	MG489967.1	France	2017	Tomato	2775		Severe
452	KY656826.1	French Polynesia	2016	Tomato	2781		Severe
453	KY656825.1	French Polynesia	2015	Tomato	2781		Severe
454	GU355941.1	Guatemala	2006	Tomato	2781		Severe
455	MT551616.1	India	2019	Tomato	2780		Severe
456	MT551620.1	India	2019	Tomato	2780		Severe
457	MT551617.1	India	2019	Tomato	2780		Severe
458	MT551618.1	India	2019	Tomato	2781		Severe
459	MT551621.1	India	2019	Tomato	2781		Severe
460	MT551614.1	India	2019	Tomato	2759		Severe
461	MT551619.1	India	2019	Tomato	2759		Severe
462	MT551615.1	India	2019	Tomato	2766		Severe
463	MT551611.1	India	2018	Tomato	2765		Severe
464	MT551612.1	India	2018	Tomato	2759		Severe
465	MT551613.1	India	2018	Tomato	2759		Severe

466	MT551610.1	India	2018	Tomato	2789		Severe
467	KY284012.1	India	2014	Tomato	2789		Severe
468	KY825714.1	Iran	2015	Weed	2756		Severe
469	MF536415.1	Iran	2014	Weed	2756		Severe
470	KT990213.1	Iran	2013	Tomato	2763		Severe
471	KX347164.1	Iran	2012	Tomato	2782		Severe
472	KX347165.1	Iran	2012	Tomato	2781		Severe
473	KX347166.1	Iran	2012	Tomato	2781		Severe
474	KC106649.1	Iran	2011	Tomato	2781		Severe
475	KC106647.1	Iran	2011	Weed	2781		Severe
476	KC106648.1	Iran	2011	Tomato	2781		Severe
477	KC106646.1	Iran	2011	Weed	2781		Severe
478	KC106645.1	Iran	2011	Weed	2781		Severe
479	KC106644.1	Iran	2011	Weed	2781		Severe
480	KC106641.1	Iran	2011	Weed	2781		Severe
481	KC106643.1	Iran	2011	Weed	2781		Severe
482	KC106642.1	Iran	2011	Weed	2759		Severe
483	KC106640.1	Iran	2011	Weed	2782		Severe
484	KC106638.1	Iran	2011	Weed	2782		Severe
485	KC106635.1	Iran	2011	Weed	2782		Severe
486	KC106652.1	Iran	2011	Tomato	2779		Severe
487	KC106651.1	Iran	2011	Tomato	2779		Severe
488	KC106650.1	Iran	2011	Tomato	2779		Severe
489	KC106637.1	Iran	2011	Weed	2779		Severe
490	KC106636.1	Iran	2011	Weed	2779		Severe
491	KX347161.1	Iran	2010	Tomato	2782		Severe
492	KX347160.1	Iran	2010	Tomato	2782		Severe
493	JQ231214.1	Iran	2010	Tomato	2774		Severe
494	JQ928348.1	Iran	2010	Tomato	2779		Severe
495	JQ414025.1	Iran	2010	Tomato	2780		Severe
496	JQ928346.1	Iran	2010	Tomato	2770		Severe
497	KX347163.1	Iran	2009	Tomato	2781		Severe
498	KX347162.1	Iran	2009	Tomato	2783		Severe
499	JQ928347.1	Iran	2009	Tomato	2780		Severe
500	AJ132711.1	Iran	2009	Tomato	2771		Severe
501	EU635776.1	Iran	2009	Tomato	2770		Severe

502	KX347159.1	Iran	2008	Tomato	2782		Severe
503	KX347157.1	Iran	2007	Tomato	2782		Severe
504	GU076444.1	Iran	2007	Tomato	2781		Severe
505	GU076447.1	Iran	2007	Tomato	2781		Severe
506	GU076446.1	Iran	2007	Tomato	2781		Severe
507	GU076445.1	Iran	2007	Tomato	2781		Severe
508	GU076450.1	Iran	2007	Tomato	2753		Severe
509	KX347158.1	Iran	2006	Tomato	2782		Severe
510	KX347156.1	Iran	2006	Tomato	2783		Severe
511	KX347155.1	Iran	2006	Tomato	2782		Severe
512	GU076440.1	Iran	2006	Tomato	2781		Severe
513	GU076452.1	Iran	2006	Tomato	2770		Severe
514	GU076453.1	Iran	2006	Tomato	2770		Severe
515	GU076441.1	Iran	2006	Tomato	2770		Severe
516	GU076451.1	Iran	2006	Tomato	2773		Severe
517	GU076448.1	Iran	2006	Tomato	2770		Severe
518	GU076449.1	Iran	2006	Tomato	2770		Severe
519	GU076443.1	Iran	2006	Tomato	2764		Severe
520	GU076442.1	Iran	2006	Tomato	2764		Severe
521	EU085423.2	Iran	2006	Tomato	2776		Severe
522	GU076454.1	Iran	2006	Tomato	2764		Severe
523	MT583814.1	Iraq	2022	Tomato	2770		Severe
524	JQ354991.1	Iraq	2011	Tomato	2780		Severe
525	ON254272.1	Iraq	2021	Toamto	2729 bp		Mild
526	X76319.1	Israel	2016	-	2790 bp		Mild
527	OL444941.1	Israel	2022	Tomato	2790		Severe
528	X15656.1	Isreal	1991	Tomato	2787		Severe
529	MH817479.1	Italy	2016	Tomato	2773		Severe
530	MF405078.1	Italy	2016	Tomato	2774		Severe
531	MG489968.1	Italy	2013	Tomato	2776		Severe
532	DQ144621.1	Italy	2004	Tomato	2781		Severe
533	LC642631.1	Japan	2020	Tomato	2781		Severe
534	LF707873.1	Japan	2016	Tomato	2774		Severe
535	KJ466048.1	Japan	2014	Pumpkin	2781		Severe
536	KJ466047.1	Japan	2014	Pumpkin	2781		Severe
537	KC677732.1	Japan	2011	Tomato	2784		Severe

538	KJ585666.1	Japan	2011	Common bean	2774		Severe
539	LC202091.1	Japan	2011	Texas bluebell	2768		Severe
540	AB192966.1	Japan	2005	Tomato	2781		Severe
541	AB192965.1	Japan	2005	Tomato	2781		Severe
542	AB116631.1	Japan	2004	Weed	2774		Severe
543	AB116630.1	Japan	2004	Lisianthus	2774		Severe
544	AB116629.1	Japan	2004	Tomato	2774		Severe
545	AB110217.1	Japan	2003	-	2774		Severe
546	AB921568.1	Japan	2016	Tomato	2787 bp		Mild
547	AB014346.1	Japan	2009	Tomato	2791 bp		Mild
548	AB014347.1	Japan	2009	Tomato	2787 bp		Mild
549	AB116632.1	Japan	2010	Tomato	2791 bp		Mild
550	AB116633.1	Japan	2010	Tomato	2787 bp		Mild
551	AB116634.1	Japan	2010	Tomato	2787 bp		Mild
552	AB116635.1	Japan	2010	Tomato	2787 bp		Mild
553	AB116636.1	Japan	2010	Tomato	2787 bp		Mild
554	AB439841.1	Japan	2009	-	2781 bp		Mild
555	AB363566.1	Japan	2007	Toamto	2780 bp		Mild
556	AB439842.1	Japan	2009	-	2787 bp		Mild
557	LC790472.1	Japan	2006	Toamto	2781 bp		Mild
558	LC790473.1	Japan	2006	Toamto	2781 bp		Mild
559	LC790474.1	Japan	2006	Toamto	2781 bp		Mild
560	LC790475.1	Japan	2006	Toamto	2774 bp		Mild
561	LC790476.1	Japan	2006	Toamto	2791 bp		Mild
562	JX444575.1	Jordan	2011	Tomato	2781		Severe
563	JX131286.1	Jordan	2011	Weed	2771		Severe
564	GQ861426.1	Jordan	2009	Tomato	2780		Severe
565	EF433426.1	Jordan	2009	Cucumber	2781		Severe
566	EF054893.1	Jordan	2006	-	2781		Severe
567	EF054894.1	Jordan	2006	Toamto	2791 bp		Mild
568	EF158044.1	Jordan	2007	Cucumber	2791 bp		Mild
569	EU143745.1	Jordan	2009	Cucumber	2791 bp		Mild
570	GQ861427.1	Jordan	2008	Toamto	2791 bp		Mild
571	PP179258.1	Korea	2023	Tomato	2773		Severe
572	PP179259.1	Korea	2023	Tomato	2773		Severe
573	PP179261.1	Korea	2023	Tomato	2773		Severe

574	PP179262.1	Korea	2023	Tomato	2773		Severe
575	PP179263.1	Korea	2023	Tomato	2773		Severe
576	PP179264.1	Korea	2023	Tomato	2773		Severe
577	PP179265.1	Korea	2023	Tomato	2773		Severe
578	PP179266.1	Korea	2023	Tomato	2773		Severe
579	PP179260.1	Korea	2023	Tomato	2773		Severe
580	PP179267.1	Korea	2023	Tomato	2773		Severe
581	PP179268.1	Korea	2023	Tomato	2773		Severe
582	PP179269.1	Korea	2023	Tomato	2773		Severe
583	PP179270.1	Korea	2023	Tomato	2773		Severe
584	PP179272.1	Korea	2023	Tomato	2773		Severe
585	PP179271.1	Korea	2023	Tomato	2773		Severe
586	PP179290.1	Korea	2023	Tomato	2774		Severe
587	PP179291.1	Korea	2023	Tomato	2774		Severe
588	PP179292.1	Korea	2023	Tomato	2774		Severe
589	PP179299.1	Korea	2023	Tomato	2774		Severe
590	PP179293.1	Korea	2023	Tomato	2774		Severe
591	PP179296.1	Korea	2023	Tomato	2774		Severe
592	PP179294.1	Korea	2023	Tomato	2774		Severe
593	PP179295.1	Korea	2023	Tomato	2774		Severe
594	PP179297.1	Korea	2023	Tomato	2774		Severe
595	PP179298.1	Korea	2023	Tomato	2774		Severe
596	OR644906.1	Korea	2022	Tomato	2773		Severe
597	OR644904.1	Korea	2022	Tomato	2773		Severe
598	OR644905.1	Korea	2022	Tomato	2773		Severe
599	OR644897.1	Korea	2022	Tomato	2773		Severe
600	OR644912.1	Korea	2022	Tomato	2773		Severe
601	OR644909.1	Korea	2022	Tomato	2773		Severe
602	OR644908.1	Korea	2022	Tomato	2773		Severe
603	OR644902.1	Korea	2022	Tomato	2773		Severe
604	OR644903.1	Korea	2022	Tomato	2773		Severe
605	OR644901.1	Korea	2022	Tomato	2773		Severe
606	OR644899.1	Korea	2022	Tomato	2773		Severe
607	OR644900.1	Korea	2022	Tomato	2773		Severe
608	OR644898.1	Korea	2022	Tomato	2773		Severe
609	OR644910.1	Korea	2022	Tomato	2773		Severe

610	OR644911.1	Korea	2022	Tomato	2773		Severe
611	OR644907.1	Korea	2022	Tomato	2773		Severe
612	OR644882.1	Korea	2022	Tomato	2773		Severe
613	OR644885.1	Korea	2022	Tomato	2773		Severe
614	OR644886.1	Korea	2022	Tomato	2773		Severe
615	OR644884.1	Korea	2022	Tomato	2774		Severe
616	OR644883.1	Korea	2022	Tomato	2773		Severe
617	OR644881.1	Korea	2022	Tomato	2773		Severe
618	ON982193.1	Korea	2021	Tomato	2773		Severe
619	ON982194.1	Korea	2021	Tomato	2773		Severe
620	ON982196.1	Korea	2021	Tomato	2773		Severe
621	ON982199.1	Korea	2021	Tomato	2773		Severe
622	ON982200.1	Korea	2021	Tomato	2773		Severe
623	ON982198.1	Korea	2021	Tomato	2773		Severe
624	ON982195.1	Korea	2021	Tomato	2773		Severe
625	ON982197.1	Korea	2021	Tomato	2773		Severe
626	ON982191.1	Korea	2021	Tomato	2773		Severe
627	ON982189.1	Korea	2021	Tomato	2773		Severe
628	ON982190.1	Korea	2021	Tomato	2773		Severe
629	ON982192.1	Korea	2021	Tomato	2773		Severe
630	KY111368.1	Korea	2016	Tomato	2774		Severe
631	KF225312.1	Korea	2012	Lisianthus	2774		Severe
632	JX961666.1	Korea	2012	Tomato	2774		Severe
633	JX961665.1	Korea	2012	Tomato	2774		Severe
634	JX961667.1	Korea	2012	Tomato	2774		Severe
635	GU126513.1	Korea	2011	Tomato	2775		Severe
636	JN183876.1	Korea	2011	Tomato	2774		Severe
637	JN183873.1	Korea	2011	Tomato	2774		Severe
638	JN183872.1	Korea	2011	-	2774		Severe
639	JN183874.1	Korea	2011	Tomato	2774		Severe
640	JN183875.1	Korea	2011	Tomato	2774		Severe
641	JN183880.1	Korea	2011	Tomato	2774		Severe
642	JN183879.1	Korea	2011	Tomato	2774		Severe
643	AB636409.1	Korea	2011	Tomato	2774		Severe
644	AB636411.1	Korea	2011	Tomato	2774		Severe
645	HM856917.1	Korea	2010	-	2774		Severe

646	HM856913.1	Korea	2010	-	2774		Severe
647	HM856911.1	Korea	2010	-	2774		Severe
648	HM856914.1	Korea	2010	-	2774		Severe
649	HM856912.1	Korea	2010	-	2774		Severe
650	HM856915.1	Korea	2010	-	2774		Severe
651	HM856909.1	Korea	2010	-	2774		Severe
652	HM856919.1	Korea	2010	-	2774		Severe
653	JQ013091.1	Korea	2009	Tomato	2774		Severe
654	JQ013090.1	Korea	2009	Tomato	2774		Severe
655	JQ013089.1	Korea	2009	Tomato	2774		Severe
656	GU325634.1	Korea	2009	Tomato	2774		Severe
657	GQ141873.1	Korea	2009	Tomato	2774		Severe
658	JN680149.1	Korea	2008	Tomato	2774		Severe
659	JN680150.1	Korea	2008	Tomato	2774		Severe
660	HM130912.1	Korea	2008	Tomato	2774		Severe
661	JX961668.1	Korea	2012	Tomato	2781 bp		Mild
662	JX961669.1	Korea	2012	Tomato	2781 bp		Mild
663	AB613208.1	Korea	2010	Toamto	2781 bp		Mild
664	AB613209.1	Korea	2010	Toamto	2781 bp		Mild
665	AB636264.1	Korea	2011	Toamto	2781 bp		Mild
666	AB636410.1	Korea	2011	Toamto	2781 bp		Mild
667	AB636412.1	Korea	2011	Toamto	2781 bp		Mild
668	AB669434.1	Korea	2011	Toamto	2781 bp		Mild
669	GU325632.1	Korea	2009	Toamto	2781 bp		Mild
670	GU325633.1	Korea	2009	Toamto	2781 bp		Mild
671	HM130913.1	Korea	2008	Toamto	2781 bp		Mild
672	HM130914.1	Korea	2008	Toamto	2781 bp		Mild
673	HM856873.1	Korea	2008	Toamto	2786 bp		Mild
674	HM856910.1	Korea	2010	-	2781 bp		Mild
675	HM856916.1	Korea	2010	-	2781 bp		Mild
676	HM856918.1	Korea	2010	-	2781 bp		Mild
677	JN183877.1	Korea	2011	Toamto	2781 bp		Mild
678	JN183878.1	Korea	2011	Toamto	2781 bp		Mild
679	ON982201.1	Korea	2021	Tomato	2791 bp		Mild
680	ON982202.1	Korea	2021	Tomato	2791 bp		Mild
681	ON982203.1	Korea	2021	Tomato	2791 bp		Mild

682	ON982204.1	Korea	2021	Tomato	2791 bp		Mild
683	ON982205.1	Korea	2021	Tomato	2791 bp		Mild
684	ON982206.1	Korea	2021	Tomato	2791 bp		Mild
685	ON982207.1	Korea	2021	Tomato	2791 bp		Mild
686	ON982208.1	Korea	2021	Tomato	2791 bp		Mild
687	ON982209.1	Korea	2021	Tomato	2791 bp		Mild
688	ON982210.1	Korea	2021	Tomato	2791 bp		Mild
689	ON982211.1	Korea	2021	Tomato	2791 bp		Mild
690	ON982212.1	Korea	2021	Tomato	2791 bp		Mild
691	ON982213.1	Korea	2021	Tomato	2791 bp		Mild
692	ON982214.1	Korea	2021	Tomato	2791 bp		Mild
693	OR644913.1	Korea	2022	Tomato	2791 bp		Mild
694	OR644914.1	Korea	2022	Tomato	2791 bp		Mild
695	OR644915.1	Korea	2022	Tomato	2791 bp		Mild
696	OR644916.1	Korea	2022	Tomato	2791 bp		Mild
697	OR644917.1	Korea	2022	Tomato	2791 bp		Mild
698	OR644918.1	Korea	2022	Tomato	2791 bp		Mild
699	OR644919.1	Korea	2022	Tomato	2791 bp		Mild
700	OR644920.1	Korea	2022	Tomato	2791 bp		Mild
701	OR644921.1	Korea	2022	Tomato	2791 bp		Mild
702	OR644922.1	Korea	2022	Tomato	2791 bp		Mild
703	OR644923.1	Korea	2022	Tomato	2791 bp		Mild
704	OR644925.1	Korea	2022	Tomato	2791 bp		Mild
705	OR644924.1	Korea	2022	Tomato	2791 bp		Mild
706	OR644926.1	Korea	2022	Tomato	2791 bp		Mild
707	OR644927.1	Korea	2022	Tomato	2791 bp		Mild
708	OR644928.1	Korea	2022	Tomato	2791 bp		Mild
709	OR644929.1	Korea	2022	Tomato	2791 bp		Mild
710	OR644930.1	Korea	2022	Tomato	2791 bp		Mild
711	OR644931.1	Korea	2022	Tomato	2791 bp		Mild
712	OR644932.1	Korea	2022	Tomato	2791 bp		Mild
713	OR644933.1	Korea	2022	Tomato	2791 bp		Mild
714	OR644934.1	Korea	2022	Tomato	2791 bp		Mild
715	OR644935.1	Korea	2022	Tomato	2791 bp		Mild
716	OR644936.1	Korea	2022	Tomato	2791 bp		Mild
717	OR644937.1	Korea	2022	Tomato	2791 bp		Mild

718	OR644938.1	Korea	2022	Tomato	2791 bp		Mild
719	OR644939.1	Korea	2022	Tomato	2791 bp		Mild
720	OR644940.1	Korea	2022	Tomato	2791 bp		Mild
721	OR644941.1	Korea	2022	Tomato	2791 bp		Mild
722	OR644942.1	Korea	2022	Tomato	2791 bp		Mild
723	PP179300.1	Korea	2023	Tomato	2774		Severe
724	PP179301.1	Korea	2023	Tomato	2774		Severe
725	OR644890.1	Korea	2022	Tomato	2774		Severe
726	OR644891.1	Korea	2022	Tomato	2774		Severe
727	OR644892.1	Korea	2022	Tomato	2774		Severe
728	OR644893.1	Korea	2022	Tomato	2804		Severe
729	OR644895.1	Korea	2022	Tomato	2774		Severe
730	OR644894.1	Korea	2022	Tomato	2774		Severe
731	OR644887.1	Korea	2022	Tomato	2774		Severe
732	OR644896.1	Korea	2022	Tomato	2774		Severe
733	OR644889.1	Korea	2022	Tomato	2772		Severe
734	OR644888.1	Korea	2022	Tomato	2774		Severe
735	ON982177.1	Korea	2021	Tomato	2774		Severe
736	ON982176.1	Korea	2021	Tomato	2774		Severe
737	ON982178.1	Korea	2021	Tomato	2774		Severe
738	ON982182.1	Korea	2021	Tomato	2774		Severe
739	ON982184.1	Korea	2021	Tomato	2774		Severe
740	ON982183.1	Korea	2021	Tomato	2774		Severe
741	ON982185.1	Korea	2021	Tomato	2774		Severe
742	ON982186.1	Korea	2021	Tomato	2774		Severe
743	ON982179.1	Korea	2021	Tomato	2774		Severe
744	ON982181.1	Korea	2021	Tomato	2774		Severe
745	ON982180.1	Korea	2021	Tomato	2774		Severe
746	ON982175.1	Korea	2021	Tomato	2774		Severe
747	ON982187.1	Korea	2021	Tomato	2774		Severe
748	ON982188.1	Korea	2021	Tomato	2774		Severe
749	KY022525.1	Kuwait	2015	-	2782		Severe
750	KR108214.1	Kuwait	2012	Tomato	2781		Severe
751	KJ830842.1	Kuwait	2012	Tomato	2781		Severe
752	KJ830841.1	Kuwait	2010	Tomato	2780		Severe
753	EF051116.1	Lebanon	2006	-	2781		Severe

754	EF185318.1	Lebanon	2006	-	2790 bp		Mild
755	HM448447.1	Mauritius	2009	Tomato	2757		Severe
756	KX347172.1	Mauritius	2009	Tomato	2780		Severe
757	KX347171.1	Mauritius	2009	Tomato	2780		Severe
758	KX347170.1	Mauritius	2009	Tomato	2781		Severe
759	KX347169.1	Mauritius	2009	Tomato	2781		Severe
760	KX347168.1	Mauritius	2009	Tomato	2781		Severe
761	KX347167.1	Mauritius	2009	Tomato	2781		Severe
762	MF945599.1	Mexico	2016	Pepper	2768		Severe
763	MF945598.1	Mexico	2016	Pepper	2768		Severe
764	KX440610.1	Mexico	2015	Pepper	2768		Severe
765	KX440609.1	Mexico	2015	Pepper	2768		Severe
766	KU836749.1	Mexico	2015	Tomato	2768		Severe
767	KX440607.1	Mexico	2015	Pepper	2768		Severe
768	KX440608.1	Mexico	2015	Pepper	2765		Severe
769	KX440606.1	Mexico	2015	Pepper	2765		Severe
770	KX427166.1	Mexico	2015	Tomato	2781		Severe
771	KU836750.1	Mexico	2015	Tomato	2781		Severe
772	JQ303121.1	Mexico	2011	Tomato	2794		Severe
773	KX440611.1	Mexico	2008	Tomato	2772		Severe
774	HM459851.1	Mexico	2008	Pepper	2781		Severe
775	EF523478.1	Mexico	2007	-	2781		Severe
776	EF210555.1	Mexico	2007	-	2781		Severe
777	FJ609655.1	Mexico	2006	Tomato	2781		Severe
778	DQ631892.1	Mexico	2006	-	2781		Severe
779	LN812978.1	Morocco	2015	Tomato	2773	Reccombinant strain	Severe
780	LN846615.1	Morocco	2014	Tomato	2781		Severe
781	LN846610.1	Morocco	2014	Tomato	2773	Recombinant strain	Severe
782	LN846606.1	Morocco	2014	Tomato	2773	Recombinant strain	Severe
783	LN846604.1	Morocco	2014	Tomato	2773	Recombinant strain	Severe
784	LN846608.1	Morocco	2014	Tomato	2773	Recombinant strain	Severe
785	LN846607.1	Morocco	2014	Tomato	2773	Recombinant strain	Severe
786	LN846605.1	Morocco	2014	Tomato	2773	Recombinant strain	Severe
787	LN846603.1	Morocco	2014	Tomato	2773	Recombinant strain	Severe
788	LN846614.1	Morocco	2013	Tomato	2781		Severe
789	LN846613.1	Morocco	2013	Tomato	2780		Severe

790	LN846600.1	Morocco	2013	Tomato	2773	Recombinant strain	Severe
791	LN846609.1	Morocco	2013	Tomato	2773	Recombinant strain	Severe
792	LN846599.1	Morocco	2012	Tomato	2773	Recombinant strain	Severe
793	LN846602.1	Morocco	2012	Tomato	2773	Recombinant strain	Severe
794	LN846601.1	Morocco	2012	Tomato	2773	Recombinant strain	Severe
795	LN831187.1	Morocco	2010	Tomato	2773	Recombinant strain	Severe
796	EF060196.1	Morocco	2008	-	2781		Severe
797	LN846616.1	Morocco	2003	Tomato	2781		Severe
798	LN846617.1	Morocco	2002	Tomato	2778		Severe
799	FJ439569.1	Netherlands	2008	Tomato	2781		Severe
800	HE603246.1	New Caledonia	2010	Tomato	2780		Severe
801	HE603242.1	New Caledonia	2010	Tomato	2780		Severe
802	HE603244.1	New Caledonia	2010	Tomato	2780		Severe
803	HE603243.1	New Caledonia	2010	Tomato	2780		Severe
804	HE603241.1	New Caledonia	2010	Tomato	2780		Severe
805	MH745751.1	Oman	2017	Papaya	2753		Severe
806	MH745750.1	Oman	2017	Poinsettia	2766		Severe
807	MG970362.1	Oman	2016	Common bean	2766		Severe
808	MK757240.1	Oman	2016	Tomato	2768		Severe
809	MK757239.1	Oman	2016	Tomato	2768		Severe
810	MK757238.1	Oman	2016	Tomato	2767		Severe
811	MK757243.1	Oman	2016	Tomato	2755		Severe
812	MK757242.1	Oman	2016	Tomato	2766		Severe
813	MK757241.1	Oman	2016	Tomato	2766		Severe
814	LN680631.1	Oman	2014	Tomato	2790		Severe
815	LN680632.1	Oman	2014	Tomato	2776		Severe
816	LN680630.1	Oman	2014	Tomato	2780		Severe
817	HG941642.1	Oman	2013	Basil	2765		Severe
818	HG969256.1	Oman	2013	-	2770		Severe
819	HG969254.1	Oman	2013	-	2763		Severe
820	HG941646.1	Oman	2013	Tobacco	2776		Severe
821	HG969287.1	Oman	2013	-	2776		Severe
822	HG969284.1	Oman	2013	-	2776		Severe
823	HG969261.1	Oman	2013	-	2776		Severe
824	HG941645.1	Oman	2013	Tobacco	2776		Severe
825	HG969286.1	Oman	2013	-	2776		Severe

826	HG969282.1	Oman	2013	-	2776		Severe
827	HG969281.1	Oman	2013	-	2776		Severe
828	HG969269.1	Oman	2013	-	2776		Severe
829	HG969267.1	Oman	2013	-	2776		Severe
830	HG969285.1	Oman	2013	-	2776		Severe
831	HG941647.1	Oman	2013	Tobacco	2776		Severe
832	HG941640.1	Oman	2013	Squash	2776		Severe
833	HG969280.1	Oman	2013	-	2776		Severe
834	HG969283.1	Oman	2013	-	2776		Severe
835	HG941649.1	Oman	2013	Tomato	2777		Severe
836	HG969270.1	Oman	2013	-	2777		Severe
837	HG969268.1	Oman	2013	-	2777		Severe
838	HG969266.1	Oman	2013	-	2781		Severe
839	HG941650.1	Oman	2013	Tomato	2777		Severe
840	HG969279.1	Oman	2013	-	2777		Severe
841	HG969271.1	Oman	2013	-	2804		Severe
842	HG969272.1	Oman	2013	-	2784		Severe
843	HG941651.1	Oman	2013	Tomato	2779		Severe
844	HG969198.1	Oman	2013	Tomato	2780		Severe
845	HG969208.1	Oman	2013	Tomato	2779		Severe
846	HG969207.1	Oman	2013	Tomato	2777		Severe
847	KF229725.1	Oman	2012	Tomato	2790		Severe
848	KF229724.1	Oman	2012	Tomato	2790		Severe
849	KF229723.1	Oman	2012	Tomato	2791		Severe
850	KF229722.1	Oman	2012	Tomato	2763		Severe
851	KF229721.1	Oman	2012	Tomato	2763		Severe
852	KF229726.1	Oman	2012	Tomato	2784		Severe
853	HE819242.1	Oman	2011	Tomato	2763		Severe
854	HE819240.1	Oman	2011	Pepper	2778		Severe
855	HE819245.1	Oman	2011	Tomato	2774		Severe
856	JN604485.1	Oman	2011	Tomato	2776		Severe
857	JN604484.1	Oman	2011	Tomato	2777		Severe
858	HE819243.1	Oman	2011	Tomato	2779		Severe
859	HE819241.1	Oman	2011	Tomato	2766		Severe
860	JN604488.1	Oman	2011	Tomato	2767		Severe
861	JN604487.1	Oman	2011	Tomato	2767		Severe

862	JN604486.1	Oman	2011	Tomato	2767		Severe
863	DQ644565.1	Oman	2009	-	2765		Severe
864	FJ956705.1	Oman	2005	Tomato	2765		Severe
865	FJ956701.1	Oman	2005	Tomato	2765		Severe
866	FJ956702.1	Oman	2005	Tomato	2765		Severe
867	FJ956704.1	Oman	2005	Tomato	2755		Severe
868	FJ956703.1	Oman	2005	Tomato	2755		Severe
869	FJ956706.1	Oman	2005	Tomato	2770		Severe
870	MF996518.1	Pakistan	2017	Tomato	2765		Severe
871	MG210483.1	Pakistan	2017	Tomato	2759		Severe
872	MG210484.1	Pakistan	2017	Tomato	2759		Severe
873	KX710157.1	Pakistan	2015	Guar	2759		Severe
874	AF105975.1	Portugal	2004	-	2793 bp		Mild
875	AY134494.1	Puerto Rico	2004	-	2781		Severe
876	AM409201.1	Reunion	2004	Tomato	2781		Severe
877	AJ865337.2	Reunion	2007	Toamto	2791 bp		Mild
878	OL416209.1	Saudi Arabia	2023	-	2748		Severe
879	OL416210.1	Saudi Arabia	2023	-	2748		Severe
880	OL416216.1	Saudi Arabia	2023	-	2748		Severe
881	OR865128.1	Saudi Arabia	2019	Cucumber	2782		Severe
882	OR865126.1	Saudi Arabia	2019	Cucumber	2765		Severe
883	OR865127.1	Saudi Arabia	2019	Cucumber	2765		Severe
884	MG571546.1	Saudi Arabia	2017	Mentha	2785		Severe
885	MN397779.1	Saudi Arabia	2015	Tomato	2784		Severe
886	MN397780.1	Saudi Arabia	2015	Tomato	2791		Severe
887	KT728752.1	Saudi Arabia	2015	Tomato	2781		Severe
888	KT728746.1	Saudi Arabia	2015	Tomato	2781		Severe
889	KT728745.1	Saudi Arabia	2015	Cucumber	2778		Severe
890	KT728744.1	Saudi Arabia	2015	Cucumber	2779		Severe
891	KT728743.1	Saudi Arabia	2015	Cucumber	2780		Severe
892	KT355023.1	Saudi Arabia	2014	Corchorus	2790		Severe
893	KU248482.1	Saudi Arabia	2014	Ridge gourd	2788		Severe
894	KF435137.1	Saudi Arabia	2013	Tomato	2771		Severe
895	KC845301.1	Saudi Arabia	2013	Tomato	2780		Severe
896	KF435136.1	Saudi Arabia	2012	Pepper	2764		Severe
897	KF561125.1	Saudi Arabia	2012	Tomato	2775		Severe

898	OP428639.1	Spain	2019	Tomato	2773		Severe
899	NC_004005.1	Spain	2018	Pepper	2781		Severe
900	MH931769.1	Spain	2017	Tomato	2780		Severe
901	MG715488.1	Spain	2017	Tomato	2780		Severe
902	MH931768.1	Spain	2017	Tomato	2773	Recombinant strain	Severe
903	MH931767.1	Spain	2017	Tomato	2773	Recombinant strain	Severe
904	MH931766.1	Spain	2017	Tomato	2773	Recombinant strain	Severe
905	OP428640.1	Spain	2017	Tomato	2773		Severe
906	MH644778.1	Spain	2016	Tomato	2780		Severe
907	MH644781.1	Spain	2016	Tomato	2780		Severe
908	MH644779.1	Spain	2016	Tomato	2780		Severe
909	MH644783.1	Spain	2016	Tomato	2780		Severe
910	MH644785.1	Spain	2016	Tomato	2780		Severe
911	MH644780.1	Spain	2016	Tomato	2781		Severe
912	MH644782.1	Spain	2016	Tomato	2781		Severe
913	MH644784.1	Spain	2016	Tomato	2781		Severe
914	MH680948.1	Spain	2015	Tomato	2780		Severe
915	MH680955.1	Spain	2015	Tomato	2780		Severe
916	MH680954.1	Spain	2015	Tomato	2780		Severe
917	MH680951.1	Spain	2015	Tomato	2780		Severe
918	MH680956.1	Spain	2015	Tomato	2781		Severe
919	MH680958.1	Spain	2015	Tomato	2780		Severe
920	MH644787.1	Spain	2015	Tomato	2780		Severe
921	MH680947.1	Spain	2015	Tomato	2780		Severe
922	MH680957.1	Spain	2015	Tomato	2781		Severe
923	MH680949.1	Spain	2015	Tomato	2781		Severe
924	MH680953.1	Spain	2015	Tomato	2781		Severe
925	MH680952.1	Spain	2015	Tomato	2781		Severe
926	MH680950.1	Spain	2015	Tomato	2781		Severe
927	MH644786.1	Spain	2015	Tomato	2781		Severe
928	MH644788.1	Spain	2015	Tomato	2781		Severe
929	AJ489258.1	Spain	2009	Pepper	2781		Severe
930	KC953602.1	Spain	2003	Pepper	2781		Severe
931	AF071228.1	Spain	2004	-	2791 bp		Mild
932	AJ519441.1	Spain	2009	Tobacco	2790 bp		Mild
933	AY044138.1	Sudan	2006	-	2780		Severe

934	HF548825.1	Sweden	2009	Toamto	2798 bp		Mild
935	ON864371.1	Syria	2019	Whitefly	2787		Severe
936	ON864372.1	Syria	2019	Tomato	2787		Severe
937	ON864373.1	Syria	2019	Whitefly	2787		Severe
938	ON864374.1	Syria	2019	Tomato	2787		Severe
939	ON864375.1	Syria	2019	Whitefly	2787		Severe
940	ON864376.1	Syria	2019	Tomato	2787		Severe
941	ON864377.1	Syria	2019	Whitefly	2791 bp		Mild
942	ON864378.1	Syria	2019	Toamto	2791 bp		Mild
943	ON864379.1	Syria	2019	Whitefly	2791 bp		Mild
944	ON864380.1	Syria	2019	Toamto	2791 bp		Mild
945	ON864381.1	Syria	2019	Whitefly	2791 bp		Mild
946	ON864382.1	Syria	2019	Toamto	2791 bp		Mild
947	KY996463.1	Trinidad and Tobago	2016	Cowpea	2752		Severe
948	KY996462.1	Trinidad and Tobago	2016	Cowpea	2754		Severe
949	KY996461.1	Trinidad and Tobago	2016	Cowpea	2756		Severe
950	KY996460.1	Trinidad and Tobago	2016	Cowpea	2755		Severe
951	KY996459.1	Trinidad and Tobago	2016	Cowpea	2756		Severe
952	KY996458.1	Trinidad and Tobago	2016	Cowpea	2754		Severe
953	KY996455.1	Trinidad and Tobago	2016	Cowpea	2754		Severe
954	KY996454.1	Trinidad and Tobago	2016	Cowpea	2754		Severe
955	KY996457.1	Trinidad and Tobago	2016	Cowpea	2755		Severe
956	KY996456.1	Trinidad and Tobago	2016	Cowpea	2757		Severe
957	KU981049.1	Trinidad and Tobago	2015	Tomato	2749		Severe
958	KU981046.1	Trinidad and Tobago	2015	Tomato	2749		Severe
959	KU981048.1	Trinidad and Tobago	2015	Tomato	2752		Severe
960	KU981047.1	Trinidad and Tobago	2015	Tomato	2752		Severe
961	KU981042.1	Trinidad and Tobago	2015	Tomato	2751		Severe
962	KU981041.1	Trinidad and Tobago	2015	Tomato	2750		Severe
963	KU981040.1	Trinidad and Tobago	2015	Tomato	2752		Severe
964	KU981044.1	Trinidad and Tobago	2015	Tomato	2750		Severe
965	KU981045.1	Trinidad and Tobago	2015	Tomato	2746		Severe
966	KU981043.1	Trinidad and Tobago	2015	Tomato	2748		Severe
967	EF101929.1	Tunisia	2008	-	2781		Severe
968	AJ812277.1	Turkey	2004	Tomato	2781		Severe
969	KY810789.1	United Kingdom	2016	Tomato	2781		Severe

970	ON785706.1	USA	2021	Tomato	2777		Severe
971	ON321843.1	USA	2021	Pepper	2782		Severe
972	MW373746.1	USA	2019	Tomato	2781		Severe
973	MW373747.1	USA	2019	Tomato	2781		Severe
974	MW165297.1	USA	2019	Tomato	2752		Severe
975	MW165296.1	USA	2019	Tomato	2781		Severe
976	MW165298.1	USA	2019	Tomato	2781		Severe
977	MF669119.1	USA	2016	Tomato	2752		Severe
978	MF669117.1	USA	2016	Tomato	2752		Severe
979	MF669092.1	USA	2016	Tomato	2752		Severe
980	MF669112.1	USA	2016	Tomato	2752		Severe
981	MF669113.1	USA	2016	Tomato	2752		Severe
982	MF669089.1	USA	2016	Tomato	2752		Severe
983	KY965893.1	USA	2016	Tomato	2752		Severe
984	KY965892.1	USA	2016	Tomato	2752		Severe
985	MF669088.1	USA	2016	Tomato	2752		Severe
986	KY965889.1	USA	2016	Tomato	2752		Severe
987	MF669114.1	USA	2016	Tomato	2752		Severe
988	KY965845.1	USA	2016	Tomato	2752		Severe
989	KY965902.1	USA	2016	Tomato	2752		Severe
990	KY965900.1	USA	2016	Tomato	2752		Severe
991	KY965901.1	USA	2016	Tomato	2752		Severe
992	MF669118.1	USA	2016	Tomato	2752		Severe
993	MF669093.1	USA	2016	Tomato	2752		Severe
994	KY965866.1	USA	2016	Tomato	2752		Severe
995	KY965864.1	USA	2016	Tomato	2752		Severe
996	MF669111.1	USA	2016	Tomato	2752		Severe
997	MF669102.1	USA	2016	Tomato	2752		Severe
998	MF669101.1	USA	2016	Tomato	2752		Severe
999	MF669100.1	USA	2016	Tomato	2752		Severe
1000	MF669094.1	USA	2016	Tomato	2752		Severe
1001	KY965905.1	USA	2016	Tomato	2752		Severe
1002	KY965904.1	USA	2016	Tomato	2752		Severe
1003	KY965903.1	USA	2016	Tomato	2752		Severe
1004	KY965887.1	USA	2016	Tomato	2752		Severe
1005	KY965906.1	USA	2016	Tomato	2752		Severe

1006	KY965878.1	USA	2016	Tomato	2752		Severe
1007	KY965877.1	USA	2016	Tomato	2752		Severe
1008	KY965876.1	USA	2016	Tomato	2752		Severe
1009	KY965851.1	USA	2016	Tomato	2752		Severe
1010	KY965850.1	USA	2016	Tomato	2752		Severe
1011	MF669097.1	USA	2016	Tomato	2752		Severe
1012	MF669095.1	USA	2016	Tomato	2752		Severe
1013	KY965849.1	USA	2016	Tomato	2752		Severe
1014	MF669110.1	USA	2016	Tomato	2752		Severe
1015	KY965880.1	USA	2016	Tomato	2752		Severe
1016	KY965879.1	USA	2016	Tomato	2752		Severe
1017	MF669116.1	USA	2016	Tomato	2752		Severe
1018	MF669103.1	USA	2016	Tomato	2752		Severe
1019	MF669109.1	USA	2016	Tomato	2752		Severe
1020	MF669108.1	USA	2016	Tomato	2752		Severe
1021	MF669098.1	USA	2016	Tomato	2752		Severe
1022	MF669099.1	USA	2016	Tomato	2752		Severe
1023	MF669090.1	USA	2016	Tomato	2752		Severe
1024	KY965888.1	USA	2016	Tomato	2752		Severe
1025	KY965896.1	USA	2016	Tomato	2752		Severe
1026	KY965911.1	USA	2016	Tomato	2752		Severe
1027	KY965910.1	USA	2016	Tomato	2752		Severe
1028	KY965909.1	USA	2016	Tomato	2752		Severe
1029	KY965912.1	USA	2016	Tomato	2752		Severe
1030	KY965884.1	USA	2016	Tomato	2752		Severe
1031	KY965883.1	USA	2016	Tomato	2752		Severe
1032	KY965882.1	USA	2016	Tomato	2752		Severe
1033	MF669104.1	USA	2016	Tomato	2752		Severe
1034	MF669107.1	USA	2016	Tomato	2752		Severe
1035	MF669106.1	USA	2016	Tomato	2752		Severe
1036	KY965867.1	USA	2016	Tomato	2752		Severe
1037	KY965868.1	USA	2016	Tomato	2752		Severe
1038	KY965865.1	USA	2016	Tomato	2752		Severe
1039	MF669105.1	USA	2016	Tomato	2752		Severe
1040	KY965908.1	USA	2016	Tomato	2752		Severe
1041	KY965899.1	USA	2016	Tomato	2752		Severe

1042	KY965881.1	USA	2016	Tomato	2752		Severe
1043	KY965885.1	USA	2016	Tomato	2752		Severe
1044	KY965848.1	USA	2016	Tomato	2752		Severe
1045	KY965847.1	USA	2016	Tomato	2752		Severe
1046	KY965846.1	USA	2016	Tomato	2752		Severe
1047	KY965898.1	USA	2016	Tomato	2751		Severe
1048	KY965897.1	USA	2016	Tomato	2751		Severe
1049	KY965890.1	USA	2016	Tomato	2752		Severe
1050	KY965872.1	USA	2016	Tomato	2752		Severe
1051	KY965854.1	USA	2016	Tomato	2752		Severe
1052	KY965853.1	USA	2016	Tomato	2752		Severe
1053	KY965852.1	USA	2016	Tomato	2752		Severe
1054	KY965869.1	USA	2016	Tomato	2752		Severe
1055	KY965907.1	USA	2016	Tomato	2752		Severe
1056	KY965875.1	USA	2016	Tomato	2752		Severe
1057	KY965873.1	USA	2016	Tomato	2752		Severe
1058	KY965874.1	USA	2016	Tomato	2752		Severe
1059	KY965895.1	USA	2016	Tomato	2752		Severe
1060	KY965894.1	USA	2016	Tomato	2752		Severe
1061	KY965891.1	USA	2016	Tomato	2751		Severe
1062	MF669115.1	USA	2016	Tomato	2752		Severe
1063	MF669091.1	USA	2016	Tomato	2752		Severe
1064	KY965856.1	USA	2016	Tomato	2752		Severe
1065	KY965836.1	USA	2016	Tomato	2752		Severe
1066	KY965834.1	USA	2016	Tomato	2752		Severe
1067	KY965835.1	USA	2016	Tomato	2752		Severe
1068	KY965863.1	USA	2016	Tomato	2752		Severe
1069	KY965886.1	USA	2016	Tomato	2752		Severe
1070	KY965842.1	USA	2016	Tomato	2753		Severe
1071	KY965841.1	USA	2016	Tomato	2753		Severe
1072	KY965840.1	USA	2016	Tomato	2753		Severe
1073	KY965871.1	USA	2016	Tomato	2752		Severe
1074	KY965870.1	USA	2016	Tomato	2752		Severe
1075	KY965857.1	USA	2016	Tomato	2752		Severe
1076	KY965855.1	USA	2016	Tomato	2752		Severe
1077	KY965843.1	USA	2016	Tomato	2752		Severe

1078	MF669096.1	USA	2016	Tomato	2752		Severe
1079	KY965844.1	USA	2016	Tomato	2752		Severe
1080	KY965861.1	USA	2016	Tomato	2751		Severe
1081	KY965860.1	USA	2016	Tomato	2752		Severe
1082	KY965859.1	USA	2016	Tomato	2751		Severe
1083	KY965858.1	USA	2016	Tomato	2752		Severe
1084	KY965839.1	USA	2016	Tomato	2751		Severe
1085	KY965837.1	USA	2016	Tomato	2751		Severe
1086	KY965838.1	USA	2016	Tomato	2751		Severe
1087	KY965862.1	USA	2016	Tomato	2753		Severe
1088	KY965923.1	USA	2016	Tomato	2752		Severe
1089	KY965921.1	USA	2016	Tomato	2752		Severe
1090	KY965922.1	USA	2016	Tomato	2752		Severe
1091	KY971343.1	USA	2016	Tomato	2752		Severe
1092	KY971342.1	USA	2016	Tomato	2752		Severe
1093	KY965920.1	USA	2016	Tomato	2752		Severe
1094	KY965918.1	USA	2016	Tomato	2752		Severe
1095	KY965919.1	USA	2016	Tomato	2752		Severe
1096	KY965916.1	USA	2016	Tomato	2752		Severe
1097	KY965915.1	USA	2016	Tomato	2752		Severe
1098	KY971372.1	USA	2016	Tomato	2752		Severe
1099	KY971348.1	USA	2016	Tomato	2752		Severe
1100	KY971341.1	USA	2016	Tomato	2752		Severe
1101	KY971339.1	USA	2016	Tomato	2752		Severe
1102	KY971351.1	USA	2016	Tomato	2752		Severe
1103	KY971347.1	USA	2016	Tomato	2752		Severe
1104	KY971346.1	USA	2016	Tomato	2752		Severe
1105	KY971352.1	USA	2016	Tomato	2752		Severe
1106	KY971349.1	USA	2016	Tomato	2752		Severe
1107	KY971344.1	USA	2016	Tomato	2752		Severe
1108	KY971338.1	USA	2016	Tomato	2752		Severe
1109	KY971345.1	USA	2016	Tomato	2752		Severe
1110	KY965914.1	USA	2016	Tomato	2752		Severe
1111	KY965913.1	USA	2016	Tomato	2752		Severe
1112	KY971350.1	USA	2016	Tomato	2752		Severe
1113	KY965917.1	USA	2016	Tomato	2752		Severe

1114	KY971340.1	USA	2016	Tomato	2752		Severe
1115	MF687351.1	USA	2015	Tomato	2752		Severe
1116	MF687350.1	USA	2015	Tomato	2752		Severe
1117	KY971367.1	USA	2015	Tomato	2752		Severe
1118	KY971359.1	USA	2015	Tomato	2752		Severe
1119	KY971354.1	USA	2015	Tomato	2752		Severe
1120	KY971363.1	USA	2015	Tomato	2752		Severe
1121	KY971362.1	USA	2015	Tomato	2752		Severe
1122	KY971355.1	USA	2015	Tomato	2752		Severe
1123	KY971368.1	USA	2015	Tomato	2752		Severe
1124	KY971361.1	USA	2015	Tomato	2752		Severe
1125	KY971371.1	USA	2015	Tomato	2754		Severe
1126	KY971370.1	USA	2015	Tomato	2752		Severe
1127	KY971365.1	USA	2015	Tomato	2752		Severe
1128	KY971358.1	USA	2015	Tomato	2752		Severe
1129	KY971357.1	USA	2015	Tomato	2752		Severe
1130	KY971356.1	USA	2015	Tomato	2752		Severe
1131	KY971353.1	USA	2015	Tomato	2752		Severe
1132	KY971360.1	USA	2015	Tomato	2752		Severe
1133	KY971364.1	USA	2015	Tomato	2752		Severe
1134	KY971366.1	USA	2015	Tomato	2752		Severe
1135	KY971369.1	USA	2015	Tomato	2752		Severe
1136	KY971337.1	USA	2015	Tomato	2752		Severe
1137	KY971336.1	USA	2015	Tomato	2752		Severe
1138	KY971332.1	USA	2015	Tomato	2752		Severe
1139	KY971335.1	USA	2015	Tomato	2752		Severe
1140	KY971334.1	USA	2015	Tomato	2752		Severe
1141	KY971333.1	USA	2015	Tomato	2752		Severe
1142	KY971331.1	USA	2015	Tomato	2752		Severe
1143	KY971330.1	USA	2015	Tomato	2753		Severe
1144	KY971329.1	USA	2015	Tomato	2753		Severe
1145	KY971325.1	USA	2015	Tomato	2752		Severe
1146	KY971324.1	USA	2015	Tomato	2752		Severe
1147	KY971322.1	USA	2015	Tomato	2752		Severe
1148	KY971321.1	USA	2015	Tomato	2752		Severe
1149	KY971320.1	USA	2015	Tomato	2752		Severe

1150	KY971323.1	USA	2015	Tomato	2752		Severe
1151	KY971327.1	USA	2015	Tomato	2752		Severe
1152	KY971326.1	USA	2015	Tomato	2781		Severe
1153	KY971328.1	USA	2015	Tomato	2781		Severe
1154	MW165293.1	USA	2014	Tomato	2752		Severe
1155	MW165294.1	USA	2014	Tomato	2752		Severe
1156	MW165295.1	USA	2014	Tomato	2752		Severe
1157	KX024650.1	USA	2014	Papaya	2781		Severe
1158	KX024647.1	USA	2014	Papaya	2781		Severe
1159	KX024648.1	USA	2014	Papaya	2781		Severe
1160	KX024649.1	USA	2014	Papaya	2781		Severe
1161	KX024646.1	USA	2014	Papaya	2781		Severe
1162	KX024641.1	USA	2014	Papaya	2781		Severe
1163	KX024640.1	USA	2014	Papaya	2781		Severe
1164	KX024642.1	USA	2014	Papaya	2781		Severe
1165	KX024639.1	USA	2014	Papaya	2781		Severe
1166	KX024645.1	USA	2014	Papaya	2781		Severe
1167	KX024644.1	USA	2014	Papaya	2781		Severe
1168	KX024643.1	USA	2014	Papaya	2781		Severe
1169	HM988987.1	USA	2010	Tomato	2781		Severe
1170	EF210554.1	USA	2010	-	2752		Severe
1171	HE603245.1	USA	2010	Tomato	2780		Severe
1172	AY530931.1	USA	2009	-	2781		Severe
1173	GU322423.2	USA	2009	Tomato	2781		Severe
1174	EF539831.1	USA	2007	-	2781		Severe
1175	EF110890.1	USA	2007	Tomato	2752		Severe
1176	GU322424.2	USA: Hawaii	2009	Tomato	2781		Severe
1177	KF477277.1	Venezuela	2009	Tomato	2791		Severe

Supplemental Table 5. Distribution of collected TYLCV genomes by country, host species, and year of collection.

Country	Host	Year	No. of genomes	Severe	Mild
Australia	Tomato	2003	1	1	0
Australia	Tomato	2006	41	41	0
Australia	Tomato	2007	3	3	0
Australia	Tomato	2009	8	8	0
Australia	Tomato	2010	7	7	0
Azerbaijan	Tomato	2014	1	1	0
China	-	2007	1	1	0
China	Amaranth	2011	1	1	0
China	Apple	2018	1	1	0
China	Asian copperleaf	2012	1	0	1
China	Bean	2016	1	1	0
China	Bitter bine	2013	1	1	0
China	Common bean	2011	1	1	0
China	Cotton	2012	1	1	0
China	Cowpea	2012	2	2	0
China	Datura	2011	1	1	0
China	Eggplant	2011	1	1	0
China	Hollyhock	2012	2	2	0
China	Pepper	2011	3	3	0
China	Pepper	2012	1	1	0
China	Pepper	2013	2	2	0
China	Pepper	2015	5	5	0
China	Perilla	2013	1	1	0
China	Pumpkin	2018	1	1	0
China	Tobacco	2022	1	1	0
China	Tomato	2006	1	1	0
China	Tomato	2007	4	4	0
China	Tomato	2008	8	8	0
China	Tomato	2009	3	3	0
China	Tomato	2010	5	5	0
China	Tomato	2011	42	42	0
China	Tomato	2012	14	14	0
China	Tomato	2013	30	30	0
China	Tomato	2014	17	17	0
China	Tomato	2015	4	4	0
China	Tomato	2016	14	14	0
China	Tomato	2017	47	47	0
China	Tomato	2018	62	62	0
China	Tomato	2019	31	31	0

China	Tomato	2020	7	7	0
China	Tomato	2021	19	19	0
China	Tomato	2022	12	12	0
China	Tomato	2023	14	14	0
China	Weed	2012	2	2	0
China	Weed	2014	1	1	0
China	Weed	2017	5	5	0
China	Whitefly	2009	1	1	0
China	Zinnia	2012	1	1	0
China	Zucchini	2019	2	2	0
Costa Rica	Tomato	2012	4	4	0
Costa Rica	Tomato	2015	2	2	0
Cuba	-	2005	1	1	0
Cuba	Tomato	2011	2	2	0
Dominican Republic	-	2003	1	1	0
Dominican Republic	Toamto	2011	2	0	2
Egypt	-	2006	1	1	0
Egypt	-	2000	1	1	0
Estonia	Tomato	2008	1	1	0
France	Tomato	2017	1	1	0
French Polynesia	Tomato	2015	1	1	0
French Polynesia	Tomato	2016	1	1	0
Guatemala	Tomato	2006	1	1	0
India	Tomato	2014	1	1	0
India	Tomato	2018	4	4	0
India	Tomato	2019	8	8	0
Iran	Tomato	2006	14	14	0
Iran	Tomato	2007	6	6	0
Iran	Tomato	2008	1	1	0
Iran	Tomato	2009	5	5	0
Iran	Tomato	2010	6	6	0
Iran	Tomato	2011	5	5	0
Iran	Tomato	2012	3	3	0
Iran	Tomato	2013	1	1	0
Iran	Weed	2011	12	12	0
Iran	Weed	2014	1	1	0
Iran	Weed	2015	1	1	0
Iraq	Toamto	2021	1	0	1
Iraq	Tomato	2011	1	1	0
Iraq	Tomato	2022	1	1	0
Israel	-	2016	1	0	1
Israel	Tomato	2022	1	1	0
Isreal	Tomato	1991	1	1	0

Italy	Tomato	2004	1	1	0
Italy	Tomato	2013	1	1	0
Italy	Tomato	2016	2	2	0
Japan	-	2009	2	0	2
Japan	-	2003	1	1	0
Japan	Common bean	2011	1	1	0
Japan	Lisianthus	2004	1	1	0
Japan	sicon esculentum (	2009	2	0	2
Japan	sicon esculentum (	2010	5	0	5
Japan	Pumpkin	2014	2	2	0
Japan	Texas bluebell	2011	1	1	0
Japan	Toamto	2006	5	0	5
Japan	Toamto	2007	1	0	1
Japan	Tomato	2004	1	1	0
Japan	Tomato	2005	2	2	0
Japan	Tomato	2011	1	1	0
Japan	Tomato	2016	2	1	1
Japan	Tomato	2020	1	1	0
Japan	Weed	2004	1	1	0
Jordan	-	2006	1	1	0
Jordan	Cucumber	2007	1	0	1
Jordan	Cucumber	2009	2	1	1
Jordan	Toamto	2006	1	0	1
Jordan	Toamto	2008	1	0	1
Jordan	Tomato	2009	1	1	0
Jordan	Tomato	2011	1	1	0
Jordan	Weed	2011	1	1	0
Korea	-	2010	11	8	3
Korea	-	2011	1	1	0
Korea	Lisianthus	2012	1	1	0
Korea	Toamto	2008	3	0	3
Korea	Toamto	2009	2	0	2
Korea	Toamto	2010	2	0	2
Korea	Toamto	2011	6	0	6
Korea	Tomato	2008	3	3	0
Korea	Tomato	2009	5	5	0
Korea	Tomato	2011	9	9	0
Korea	Tomato	2012	5	3	2
Korea	Tomato	2016	1	1	0
Korea	Tomato	2021	40	26	14
Korea	Tomato	2022	62	32	30
Korea	Tomato	2023	27	27	0
Kuwait	-	2015	1	1	0

Kuwait	Tomato	2010	1	1	0
Kuwait	Tomato	2012	2	2	0
Lebanon	-	2006	2	1	1
Mauritius	Tomato	2009	7	7	0
Mexico	-	2006	1	1	0
Mexico	-	2007	2	2	0
Mexico	Pepper	2008	1	1	0
Mexico	Pepper	2015	5	5	0
Mexico	Pepper	2016	2	2	0
Mexico	Tomato	2006	1	1	0
Mexico	Tomato	2008	1	1	0
Mexico	Tomato	2011	1	1	0
Mexico	Tomato	2015	3	3	0
Morocco	-	2008	1	1	0
Morocco	Tomato	2002	1	1	0
Morocco	Tomato	2003	1	1	0
Morocco	Tomato	2010	1	1	0
Morocco	Tomato	2012	3	3	0
Morocco	Tomato	2013	4	4	0
Morocco	Tomato	2014	8	8	0
Morocco	Tomato	2015	1	1	0
Netherlands	Tomato	2008	1	1	0
New Caledonia	Tomato	2010	5	5	0
Oman	-	2009	1	1	0
Oman	-	2013	19	19	0
Oman	Basil	2013	1	1	0
Oman	Common bean	2016	1	1	0
Oman	Papaya	2017	1	1	0
Oman	Pepper	2011	1	1	0
Oman	Poinsettia	2017	1	1	0
Oman	Squash	2013	1	1	0
Oman	Tobacco	2013	3	3	0
Oman	Tomato	2005	6	6	0
Oman	Tomato	2011	9	9	0
Oman	Tomato	2012	6	6	0
Oman	Tomato	2013	6	6	0
Oman	Tomato	2014	3	3	0
Oman	Tomato	2016	6	6	0
Pakistan	Guar	2015	1	1	0
Pakistan	Tomato	2017	3	3	0
Portugal	-	2004	1	0	1
Puerto Rico	-	2004	1	1	0
Reunion	Toamto	2007	1	0	1

Reunion	Tomato	2004	1	1	0
Saudi Arabia	-	2023	3	3	0
Saudi Arabia	Corchorus	2014	1	1	0
Saudi Arabia	Cucumber	2015	3	3	0
Saudi Arabia	Cucumber	2019	3	3	0
Saudi Arabia	Mentha	2017	1	1	0
Saudi Arabia	Pepper	2012	1	1	0
Saudi Arabia	Ridge gourd	2014	1	1	0
Saudi Arabia	Tomato	2012	1	1	0
Saudi Arabia	Tomato	2013	2	2	0
Saudi Arabia	Tomato	2015	4	4	0
Spain	-	2004	1	0	1
Spain	Pepper	2003	1	1	0
Spain	Pepper	2009	1	1	0
Spain	Pepper	2018	1	1	0
Spain	Tobacco	2009	1	0	1
Spain	Tomato	2015	15	15	0
Spain	Tomato	2016	8	8	0
Spain	Tomato	2017	6	6	0
Spain	Tomato	2019	1	1	0
Sudan	-	2006	1	1	0
Sweden	Toamto	2009	1	0	1
Syria	Bemisia tabaci	2019	3	0	3
Syria	Toamto	2019	3	0	3
Syria	Tomato	2019	3	3	0
Syria	Whitefly	2019	3	3	0
Trinidad and Tobago	Cowpea	2016	10	10	0
Trinidad and Tobago	Tomato	2015	10	10	0
Tunisia	-	2008	1	1	0
Turkey	Tomato	2004	1	1	0
United Kingdom	Tomato	2016	1	1	0
USA	-	2009	1	1	0
USA	-	2010	1	1	0
USA	-	2007	1	1	0
USA	Papaya	2014	12	12	0
USA	Pepper	2021	1	1	0
USA	Tomato	2007	1	1	0
USA	Tomato	2009	1	1	0
USA	Tomato	2010	2	2	0
USA	Tomato	2014	3	3	0
USA	Tomato	2015	39	39	0
USA	Tomato	2016	138	138	0
USA	Tomato	2019	5	5	0

USA	Tomato	2021	1	1	0
USA: Hawaii	Tomato	2009	1	1	0
Venezuela	Tomato	2009	1	1	0
		**Total**	**1177**	**1078**	**99**
